# PK-Informed Microphysiological Systems: From Dynamic Dosing to Quantitative In Vitro–In Vivo Translation

**DOI:** 10.3390/pharmaceutics18091117

**Published:** 2026-09-04

**Authors:** Su Jeong Kang, Sunghyun Bong, Min Jeong Jo, Jae Min Lee, Moon Sup Yoon, Seonmin Park, Yeseung Lee, Yuseon Shin, Hye Jin Lee, Chun-Woong Park, Dae Hwan Shin

**Affiliations:** 1 College of Pharmacy, Chungbuk National University, Cheongju 28160, Republic of Korea; 2 Korea Animal Replacement Experiment Co., Ltd. (KARE), Cheongju 28160, Republic of Korea; 3 Chungbuk National University Hospital, Chungbuk National University, Cheongju 28644, Republic of Korea

**Keywords:** physiologically based pharmacokinetic modeling, microphysiological systems, organ-on-chip, PK–PD modeling, dynamic drug exposure, drug disposition, in vitro–in vivo translation

## Abstract

Conventional in vitro drug evaluation relies largely on static concentration–response assays that fail to reproduce the dynamic pharmacokinetic (PK) profiles observed in vivo, contributing to the gap between preclinical findings and clinical outcomes. Recent advances in microphysiological systems (MPSs), particularly microfluidic organ-on-chip platforms, enable programmable concentration–time profiles that more closely mimic physiological drug exposure. These PK-informed platforms allow systematic investigation of schedule dependency, time-dependent pharmacodynamics (PD), and exposure-driven efficacy under controlled flow conditions. Spatially resolved analytical approaches further reveal heterogeneous drug penetration and metabolic responses within tissues, emphasizing the importance of spatiotemporal PK–PD coupling. Integration of multi-organ and vascularized chip systems with physiologically based pharmacokinetic (PBPK) modeling increasingly supports quantitative in vitro–in vivo translation. This review outlines how PK-informed MPSs can generate dynamic in vitro exposure and response data that inform PBPK modeling, thereby supporting quantitative in vitro–in vivo translation of drug disposition and response.

## 1. Introduction

### Limitations of Static Exposure Paradigms

Conventional in vitro drug evaluation has traditionally relied on static concentration–response assays, most commonly summarized by half-maximal inhibitory concentration (IC_50_) values [1,2]. In these experiments, cells are exposed to a constant drug concentration for a fixed duration, after which viability or other endpoints are measured [3]. Although such approaches are convenient and suitable for high-throughput screening, they treat concentration as a time-invariant input and, therefore, do not capture the temporal fluctuations that define in vivo exposure [4,5]. In reality, however, drug levels in vivo fluctuate continuously over time due to absorption, distribution, metabolism, and excretion. This fundamental mismatch between constant in vitro exposure and dynamic in vivo pharmacokinetics (PK) limits the predictive value of static IC_50_ measurements [4].

In vivo drug exposure is characterized by parameters such as maximum concentration (C_max_), time to maximum concentration (T_max_), area under the concentration–time curve (AUC), and elimination half-life [4,5]. Static assays, by contrast, collapse this complexity into a single equilibrium concentration. Increasing evidence suggests that identical cumulative exposure (AUC) delivered under different temporal patterns can produce markedly distinct pharmacodynamic (PD) outcomes [6,7]. Short high-peak exposures may induce rapid cytotoxicity, whereas prolonged low-level exposure may preferentially modulate signaling pathways, alter cell-cycle progression, or promote adaptive resistance. Consequently, drug efficacy cannot be fully captured by a single concentration metric derived under constant conditions [6].

Schedule dependency further underscores this limitation. Many anticancer agents, targeted therapies, and antimicrobial drugs exhibit either concentration-dependent or time-dependent activity [6,7]. For some compounds, therapeutic response correlates more strongly with peak concentration (C_max_), whereas others depend on duration above a threshold concentration or cumulative exposure (AUC) [5]. Static dose–response curves inherently obscure these distinctions because they do not reproduce physiologically relevant concentration–time profiles [2]. This contributes directly to the translational gap between preclinical screening and clinical performance, where compounds demonstrating potent static activity frequently fail to replicate efficacy in vivo, and vice versa [8,9].

Recent advances in microfluidic technologies have enabled the precise control of time-varying drug exposure in vitro [10,11]. By modulating flow rates and inlet compositions, microphysiological systems (MPSs) can generate programmable concentration–time profiles, including pulsatile, stepwise, or exponentially decaying exposures that resemble in vivo PK [12]. Under such dynamic conditions, cellular responses often diverge significantly from those observed in conventional static assays, revealing schedule-dependent effects that remain hidden in equilibrium-based measurements [13]. These observations challenge the sufficiency of traditional IC_50_ paradigms and highlight the need for time-resolved pharmacological evaluation [13].

Building upon these developments, the concept of micro-PK–PD has emerged as a framework for quantitatively linking dynamic drug concentrations with biological responses within MPSs [14]. Rather than treating drug concentration as a static input variable, micro-PK–PD approaches integrate real-time or programmed exposure profiles with downstream endpoints, such as viability, apoptosis, metabolic reprogramming, and biomarker expression [15]. This enables characterization of exposure thresholds, temporal delays between concentration changes and effect, nonlinear response dynamics, and adaptive feedback mechanisms [16]. By combining fluidic engineering with quantitative modeling, micro-PK–PD systems shift the experimental paradigm from equilibrium-based potency metrics toward exposure-driven, time-resolved pharmacology [17]. In doing so, they establish a mechanistic bridge between in vitro experimentation and in vivo PK reality, providing a foundation for more reliable in vitro–in vivo translation.

For this narrative review, relevant literature was identified from PubMed, Web of Science, Scopus, and Google Scholar, focusing primarily on studies published between 2010 and 2026. Representative studies were selected based on their relevance to PK-informed microphysiological systems, dynamic exposure control, PK–PD analysis, PBPK integration, and translational drug development, and foundational earlier studies were included where necessary to define key concepts.

**Figure 1:**
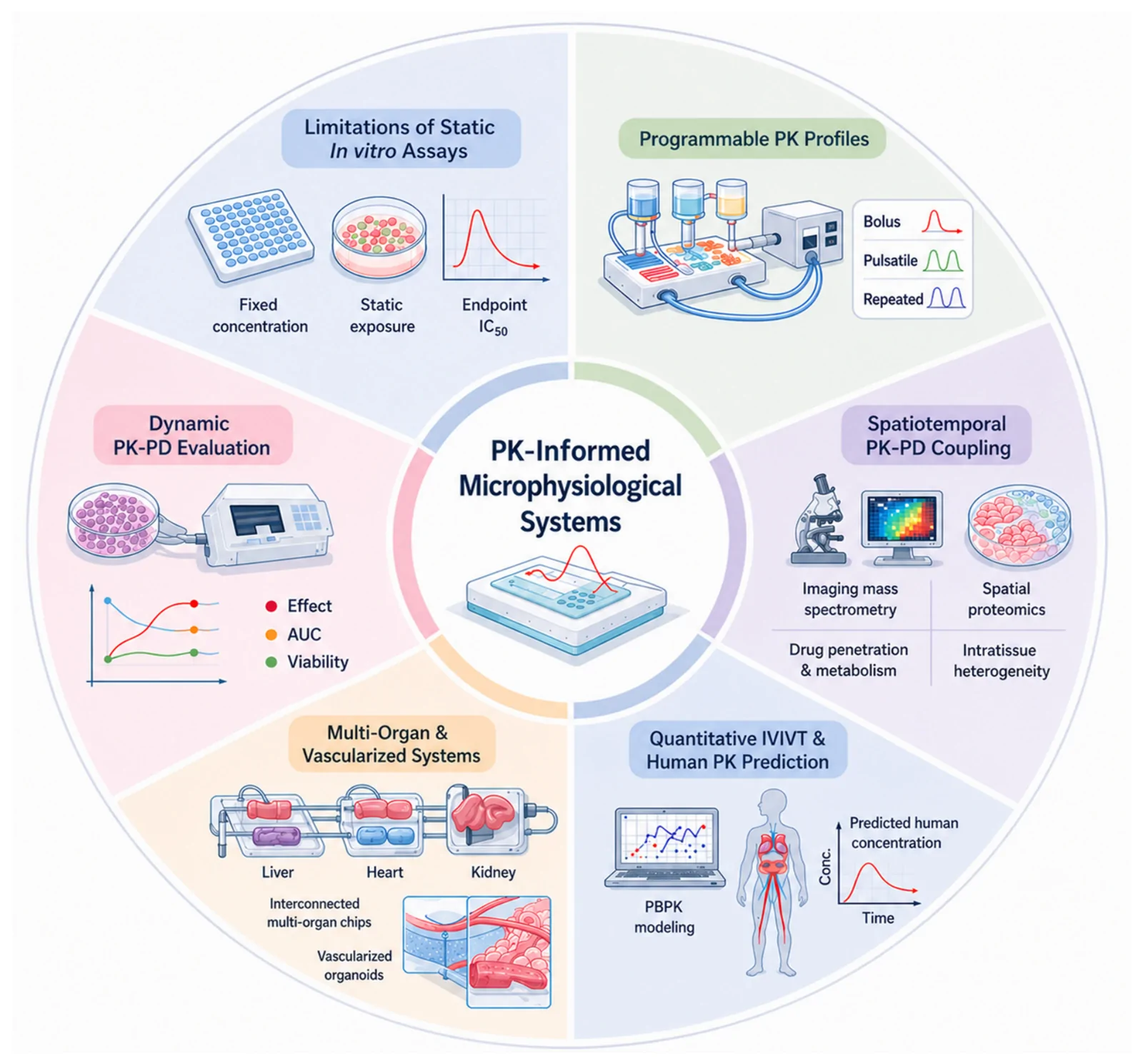
PK-informed microphysiological systems for quantitative drug translation. Schematic overview of the transition from static in vitro drug evaluation to PK-informed microphysiological systems. Programmable microfluidic platforms enable dynamic concentration–time profiles, spatiotemporal PK–PD analysis, and multi-organ or vascularized reconstruction of drug disposition. Integration with PBPK modeling and quantitative in vitro–in vivo translation (IVIVT) supports improved prediction of human pharmacokinetics and therapeutic response. (https://BioRender.com).

In this review, we discuss how PK-informed MPSs support PBPK-guided in vitro–in vivo translation by generating dynamic concentration–time profiles, exposure–response relationships, and organ-level transport or clearance parameters under controlled experimental conditions (Figure 1) [18,19]. We first examine the limitations of static exposure paradigms and review engineering strategies for reproducing dynamic PK profiles in vitro. We then discuss micro-PK–PD coupling, spatial and microenvironmental drug distribution, intestinal and multi-organ MPSs, and vascularized organ-chip platforms. Finally, we examine how these experimental systems can be integrated with PBPK modeling to improve mechanistic interpretation, parameterization, and translational prediction in drug development.

## 2. Engineering Dynamic PK Profiles In Vitro

### 2.1. Adjustable-Volume and Perfusion-Based Systems

**Figure 2:**
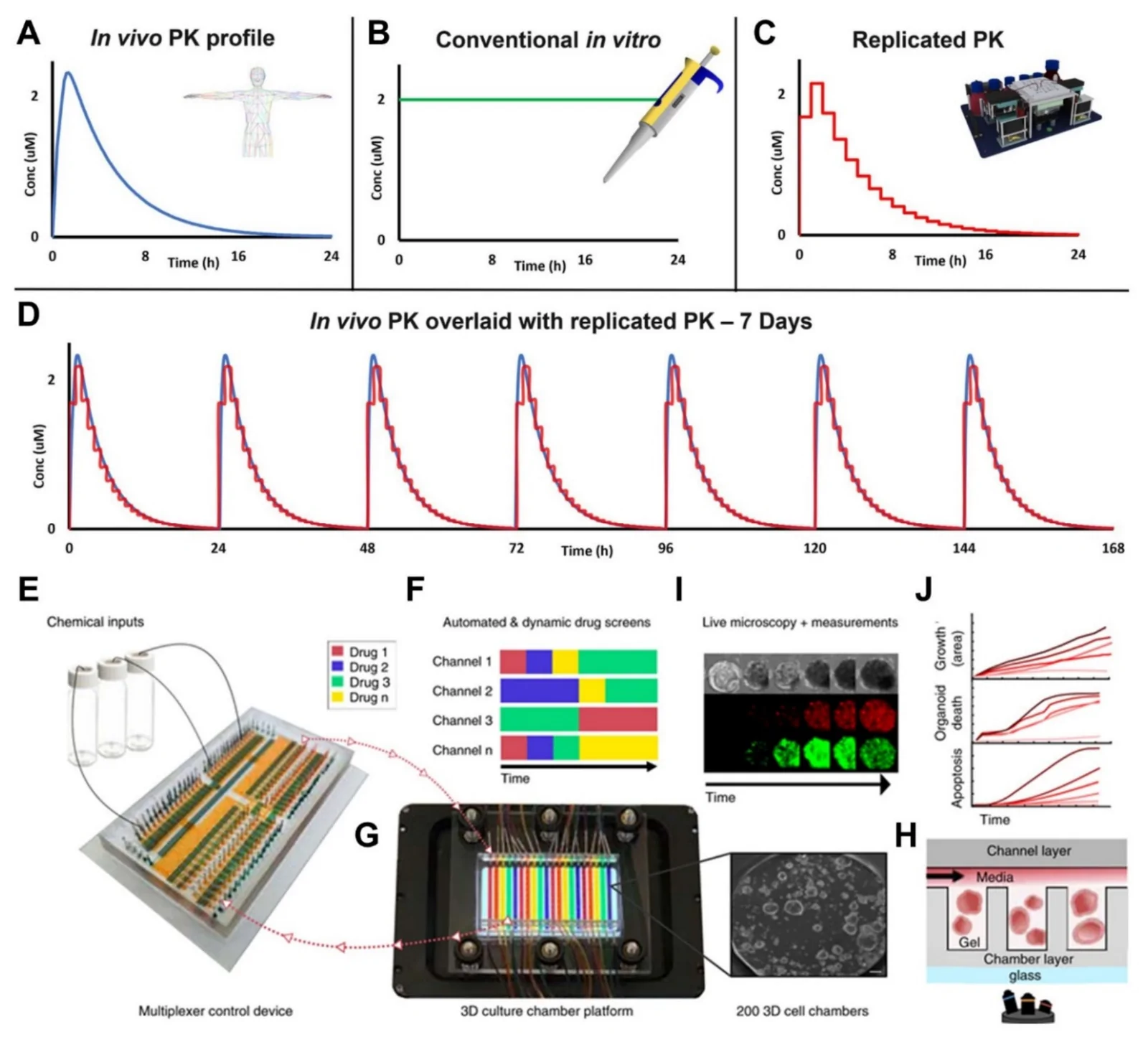
Reconstruction of dynamic in vivo-like pharmacokinetic profiles using microfluidic platforms. (**A**) Representative in vivo pharmacokinetic (PK) profile showing time-dependent drug concentration changes following administration. (**B**) Conventional in vitro exposure under static conditions, where drug concentration remains constant over time. (**C**) Replicated PK profile generated using a programmable microfluidic system, enabling stepwise approximation of in vivo-like concentration decay. (**D**) Overlay of in vivo PK (blue) and replicated PK (red) profiles over multiple dosing cycles (7 days), demonstrating high-fidelity temporal reproduction. (**E**) Schematic of multiplexed chemical input system enabling controlled delivery of multiple drug streams. (**F**) Microfluidic 3D culture chamber platform for dynamic drug exposure experiments. (**G**) Cross-sectional schematic of the culture chamber illustrating channel architecture and gel-based cell compartment. (**H**) Programmable multi-channel drug input patterns for automated and dynamic drug screening. (**I**) Real-time live-cell imaging and measurement of cellular responses under dynamic exposure conditions. (**J**) Representative quantitative outputs, including growth, organoid size, and apoptosis dynamics over time. The figure summarizes published experimental examples of dynamic PK-mimicking exposure in microfluidic culture systems. Reproduced under terms of the CC-BY license. References [40,43], Copyright 2022, The Authors, published by PLOS Biology; Copyright 2024, The Authors, published by Nature Communications.

An early strategy to reproduce time-varying PK profiles in vitro was the development of adjustable-volume cell culture systems [20]. In contrast to static culture formats, adjustable-volume platforms modulate drug concentration over time by altering the volume of culture medium in contact with cells, approximating first-order elimination kinetics without complex microfluidic architectures (Figure 2A) [21].

In the adjustable-volume model, cells are cultured in a chamber connected to a reservoir system that allows controlled addition or removal of medium. Increasing medium volume while maintaining a fixed drug amount decreases concentration over time, approximating exponential decay. Conversely, stepwise addition of drug-containing medium enables simulation of repeated dosing or bolus administration. This configuration enables programmable concentration–time profiles using relatively simple equipment [12,22,23].

A notable aspect of this system is its ability to approximate clinically relevant PK parameters, such as peak concentration (C_max_), half-life, and area under the curve (AUC). By calibrating the rate of medium exchange and volume expansion, investigators can tailor decay profiles to reflect specific elimination constants [20]. Experimentally measured concentrations follow the intended exponential profiles, allowing comparison between static exposures and dynamically declining concentrations under otherwise identical biological conditions [21].

Under these dynamically controlled exposures, cellular responses differ from those observed under constant concentration, even when cumulative exposure (AUC) is comparable (Figure 2B) [24,25]. Cells exposed to decaying profiles may show altered viability or signaling compared with constant exposure, indicating that temporal exposure history contributes to PD responses [24]. Introducing controlled PK variability into standard culture formats thus expands experimental interpretation beyond equilibrium-based IC_50_ measurements [25].

Although conceptually straightforward, adjustable-volume systems also involve trade-offs. Because concentration changes are achieved through bulk medium manipulation, spatial gradients and physiological flow effects are not reproduced. Diffusion and mixing constraints may also influence transition precision. These limitations led to further refinement through perfusion-based microfluidic platforms [12,22].

In contrast to bulk volume manipulation, perfusion-based microfluidic systems enable continuous control of drug delivery under defined flow conditions. These platforms integrate programmable syringe pumps with microscale culture chambers, allowing time-dependent modulation of drug concentration under constant flow [12]. Typically, at least two inlet channels—one containing drug solution and the other drug-free medium—permit dynamic dilution through controlled variation of individual flow rates [26,27]. By translating predefined or clinically informed concentration–time profiles into pump flow programs, PK exposure curves can be reconstructed at the site of cell culture (Figure 2C).

Serpentine mixing channels homogenize inlet streams, enabling rapid concentration transitions [26]. These systems accommodate both stepwise infusion patterns and smoothly varying exponential decay profiles. The achievable flow ranges are compatible with physiologically relevant interstitial shear stresses, introducing mechanical cues absent from static systems and shaping a more representative microenvironment for evaluating PK–PD relationships [12].

Validation studies confirm agreement between programmed and measured concentrations (Figure 2D) [27]. Fluorescent tracers and liquid chromatography–tandem mass spectrometry (LC–MS/MS) confirm that generated exposure curves follow intended PK trajectories, including species-specific profiles derived from preclinical or clinical data. Such flexibility permits evaluation of exposure–response relationships under human-relevant kinetic conditions within a controlled in vitro setting [12].

Perfusion-based PK–PD platforms also allow systematic examination of exposure duration, peak concentration, and cumulative exposure while holding other variables constant. Regimens designed to generate equivalent AUC values but differing peak concentrations often produce distinct cytotoxic responses, suggesting that total exposure alone does not fully explain biological outcome [28,29]. These systems enable simulation of clinically relevant dosing strategies, including fractionated dosing and continuous infusion [28].

This flexibility extends to multi-drug delivery. Multi-inlet configurations enable independent control of multiple agents, supporting analysis of schedule-dependent interactions under defined PK constraints (Figure 2E) [29]. Such control is particularly relevant in oncology and antimicrobial research, where sequence and timing influence therapeutic performance [28]. When dynamic in vivo-like exposure profiles are reproduced in perfusion systems, combination effects may differ from those observed under static conditions, emphasizing the role of temporal exposure patterns in interpreting in vitro data.

Technical considerations remain. Drug adsorption to device materials, particularly polydimethylsiloxane (PDMS), may alter effective exposure levels and requires compound-specific validation [30,31,32,33,34]. Accurate reproduction of target PK curves depends on appropriate modeling tools to convert concentration–time objectives into pump flow programs, along with analytical verification of achieved exposure profiles [26,27].

Further refinement has emerged through automated microfluidic platforms designed for dynamic and extended PK simulation [35,36]. In these systems, fluid delivery, mixing, and timing sequences are integrated with computerized control modules, enabling predefined concentration–time programs to be executed without continuous manual intervention [37]. Automated reservoir switching, synchronized multi-pump coordination, and real-time flow adjustment support generation of complex exposure patterns beyond simple exponential decay or stepwise infusion (Figure 2F) [38,39].

Automated systems may incorporate feedback-controlled fluidic architectures, including calibration loops or sensor integration that adjust delivery parameters during operation [39]. This reduces variability associated with pump drift or minor flow fluctuations, which becomes increasingly relevant during prolonged simulations extending over several days [38].

In addition to temporal precision, automated platforms support multiplexed experimental configurations. Parallel culture chambers within a single device can be exposed to distinct PK regimens under identical environmental conditions, enabling direct comparison of bolus versus continuous infusion, high-peak versus low-sustained exposure, or sequential versus overlapping delivery (Figure 2G,H) [40,41]. Digital control combined with microfluidic routing networks allows rapid reprogramming of exposure curves without altering device hardware [39].

Automated systems also reduce manual medium exchange steps that may perturb cellular microenvironments. Continuous, precisely timed perfusion minimizes abrupt concentration shifts and mechanical disturbances that could influence stress responses independently of drug action. Such stability becomes particularly relevant in long-duration experiments where repeated manual intervention introduces variability [36,40].

The expanded control afforded by automation enables modeling of more intricate PK scenarios, including multi-compartment transfer dynamics or clinically derived dosing schedules [39]. Integration with computational tools facilitates conversion of plasma concentration curves into programmable microfluidic instructions, linking PK modeling with experimental execution and extending temporal and quantitative control over in vitro exposure (Figure 2I,J). These engineering strategies provide the technical foundation for reproducing physiologically relevant exposure dynamics within PK-informed MPSs, where drug transport, tissue architecture, and PK variability intersect [42].

As a representative drug development example, programmable microfluidic PK replication has been used to reproduce in vivo PK profiles of oncology compounds and compare PK-mimicking exposure with conventional fixed-dose treatment. Such studies showed that dynamic exposure profiles can improve the rank-order prediction of in vivo antitumor efficacy compared with static exposure conditions, as illustrated by the experimental microfluidic platforms summarized in Figure 2 [43].

### 2.2. Programmable and Quantitative PK Curve Replication

Beyond dynamic perfusion systems, a key advance is the quantitative replication of predefined PK curves in microfluidic environments. These systems reproduce experimentally or clinically measured concentration–time trajectories with high fidelity [44]. In this framework, the in vitro platform functions not merely as a dynamic exposure device, but as a programmable PK emulator [43].

**Figure 3:**
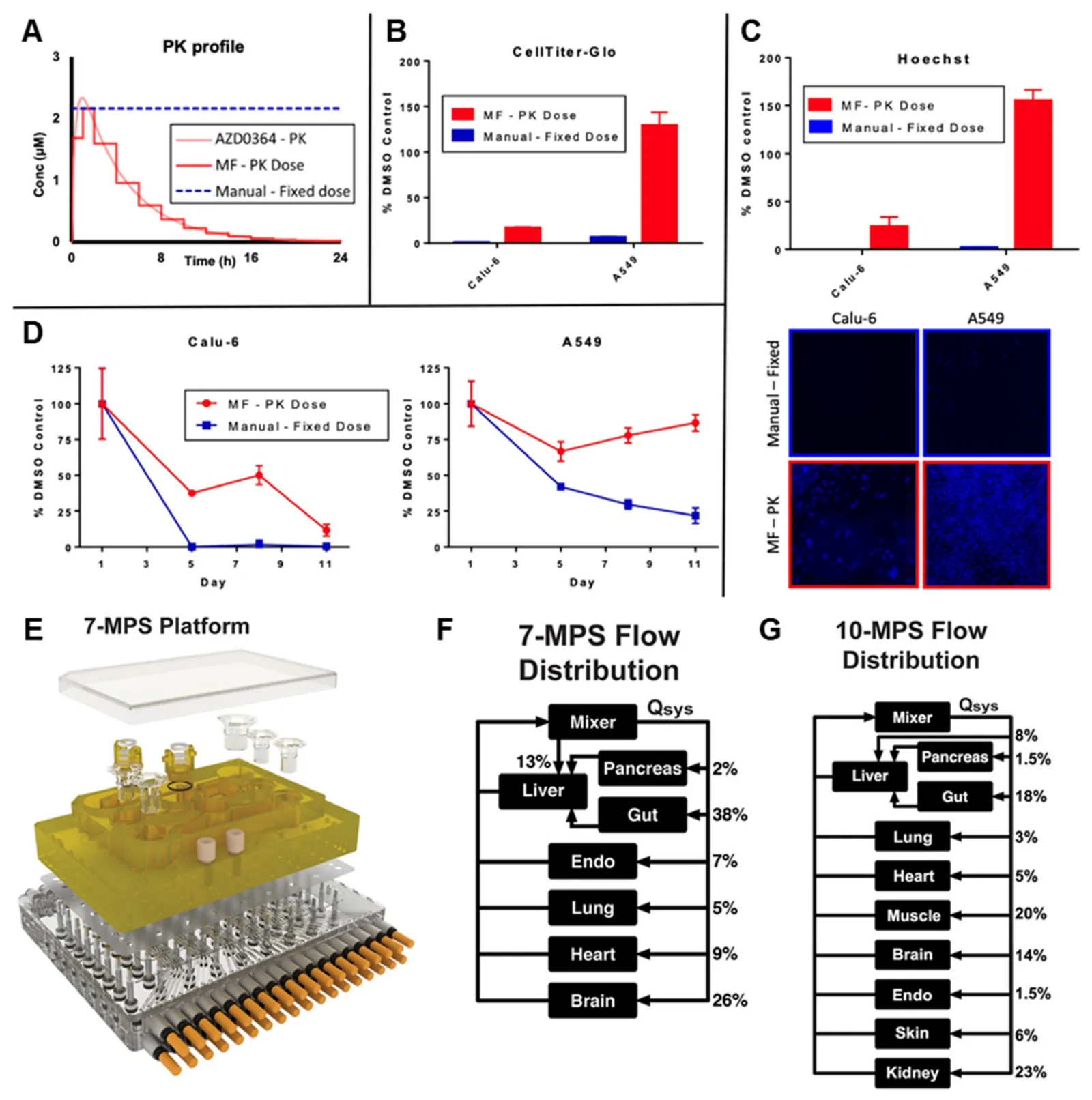
Functional impact of dynamic PK replication and extension to multi-organ microphysiological systems. (**A**) Comparison of in vivo PK profile (light red), microfluidic-replicated PK profile (red), and conventional fixed-dose exposure (blue dashed line). (**B**) Cell viability (CellTiter-Glo) under dynamic PK exposure (MF–PK dose) and manual fixed-dose conditions in Calu-6 and A549 cells. (**C**) Hoechst staining analysis showing differential cellular responses between PK-mimicking and fixed-dose treatments. (**D**) Time-dependent growth inhibition profiles under repeated dosing, demonstrating distinct response dynamics between PK-replicated and constant exposure conditions. (**E**) Schematic of a 7-organ microphysiological system (7-MPS) platform integrating multiple tissue modules. (**F**) Representative flow distribution within the 7-MPS platform, indicating relative flow fractions across interconnected organ compartments. (**G**) Expanded 10-organ microphysiological system (10-MPS) with corresponding flow distribution, illustrating physiological scaling across interconnected tissue compartments. Reproduced under terms of the CC-BY license. References [43,59], Copyright 2022, The Authors, published by PLOS Biology; Copyright 2020, The Authors, published by Scientific Reports.

A representative example is the development of a microfluidic system that directly translates in vivo PK data into time-resolved in vitro concentration profiles (Figure 3A) [43]. In this approach, experimentally obtained plasma concentration–time curves are mathematically transformed into corresponding flow rate functions, which are then executed by programmable syringe pumps. By continuously adjusting the ratio of drug-containing and drug-free media under constant total flow conditions, the system reproduces the desired concentration waveform at the site of cell exposure. This strategy allows faithful reconstruction of complex PK patterns, including rapid distribution phases followed by slower elimination phases [45].

A defining characteristic of this platform is its ability to simulate multi-phase PK rather than simple mono-exponential decay. The microfluidic system incorporates mixing channels and precisely calibrated pump control to generate smooth, continuous transitions between concentration phases, thereby approximating multi-compartment PK behavior within a single culture chamber [46,47]. Analytical validation confirms that measured outlet concentrations closely match target profiles derived from animal or human studies [43,45].

This approach allows direct comparison between distinct species-specific PK patterns under identical cellular conditions. For example, mouse and human plasma profiles of the same drug can be reproduced sequentially in the same microfluidic platform, enabling evaluation of how interspecies differences in exposure kinetics influence PD response [43]. Such experiments isolate kinetic variables from biological variability, helping explain translational discrepancies between preclinical and clinical results [48].

The system also enables investigation of dosing schedule effects beyond simple AUC normalization. Because the full concentration–time waveform is reproduced, parameters such as peak timing, decay rate, and duration above active thresholds can be systematically varied. This provides a framework for dissecting the relative contributions of C_max_, exposure duration, and temporal fluctuation to observed cellular outcomes (Figure 3B,C) [45]. In contrast to static IC_50_ measurements, which assume equilibrium exposure, programmable PK replication introduces exposure histories resembling clinical dosing [43].

From an engineering perspective, accurate curve replication requires integration of mathematical modeling with microfluidic execution. Target PK curves are digitized and converted into discrete time-dependent flow rate instructions [49]. Mixing efficiency, channel geometry, and pump response time must be calibrated to minimize lag and dispersion. Independent quantification verifies that implemented concentration profiles match theoretical targets [50,51].

By bridging experimentally derived PK data and controlled in vitro exposure, programmable PK replication platforms create an intermediate layer between PK modeling and PD observation. Rather than relying solely on computational simulations or static cell-based assays, these systems allow empirical testing of model-derived dosing scenarios under tightly regulated microenvironmental conditions [48]. This capability expands the scope of PK-informed MPSs and supports more quantitative exploration of in vitro–in vivo translation [52,53].

Beyond direct reproduction of experimentally measured PK curves, further advancement has been achieved through integration of mathematical PK modeling into microfluidic exposure systems. Rather than relying solely on empirical plasma concentration data, modeling-based approaches begin with predefined PK parameters—such as clearance, volume of distribution, and compartmental transfer rates—and computationally generate theoretical concentration–time profiles that can be executed within a microfluidic platform [48]. In this configuration, the in vitro system becomes a physical realization of a PK model, enabling experimental assessment of model-predicted dosing scenarios [54].

In modeling-driven platforms, PK equations derived from one- or multi-compartment frameworks are translated into time-dependent concentration functions. These functions are discretized and converted into corresponding pump flow rate programs, allowing dynamic reconstruction of target exposure trajectories within the cell culture chamber. This process links PK modeling with experimental PD evaluation [45]. By adjusting model parameters in silico, investigators can rapidly simulate alternative elimination rates, dosing intervals, or infusion durations and subsequently implement these modified profiles in vitro without redesigning the device architecture [54].

An important implication of this integration is the ability to interrogate PD responses under model-informed exposure conditions that may not yet have been clinically tested. For example, hypothetical regimens predicted to optimize therapeutic index based on PK modeling can be executed within the microfluidic system to observe real-time cellular responses. This enables iterative refinement between modeling and experimentation, where discrepancies between predicted and observed outcomes inform subsequent parameter adjustment [54]. In this way, the microfluidic platform operates not only as a passive emulator of known PK but as an active component of model validation and hypothesis testing [55].

Model-based curve replication also facilitates mechanistic exploration of exposure–response relationships (Figure 3D). By independently varying parameters such as elimination half-life or dosing frequency while holding other variables constant, the relative contributions of temporal exposure features can be dissected with greater resolution than is feasible in animal models [48]. Furthermore, such systems permit exploration of boundary conditions, including extreme clearance scenarios or altered distribution kinetics, which may correspond to patient subpopulations with organ dysfunction or altered metabolism [54].

Translationally, integrating PK modeling with programmable microfluidics supports a more quantitative framework for in vitro–in vivo extrapolation. Rather than comparing static potency values with systemic plasma concentrations, experimentally measured cellular responses can be directly mapped onto modeled human PK profiles [52]. This alignment reduces reliance on indirect scaling assumptions and allows exposure–response relationships to be evaluated under human-relevant kinetic constraints. These datasets support refinement of PK models and identification of exposure thresholds linked to efficacy or toxicity [55].

Technically, successful implementation requires precise calibration of mixing dynamics, flow response time, and system dead volume to ensure that implemented concentration waveforms faithfully follow model predictions. Analytical verification remains essential, as minor deviations in flow rate or adsorption behavior can distort the intended PK pattern [50,51]. Nonetheless, by embedding mathematical PK frameworks within programmable microfluidic environments, modeling-based platforms extend dynamic exposure engineering into a quantitatively predictive domain, linking theoretical PK constructs with experimentally observable PD behavior [55].

Building upon single-chamber programmable PK replication, further evolution has involved the integration of multiple interconnected compartments within a unified microfluidic platform (Figure 3E). Rather than reproducing systemic PK at a single exposure site, these systems distribute drug-containing media across spatially separated microphysiological units that represent distinct tissues. In this configuration, PK behavior emerges not solely from externally programmed concentration waveforms but from intercompartmental fluid exchange that mimics physiological circulation [54,56].

In such platforms, individual tissue chambers—often representing liver, target tissue, or additional metabolically relevant organs—are linked by defined microfluidic channels that permit controlled recirculation. Flow rates are carefully scaled to approximate physiological residence times and relative tissue volumes, enabling simulation of distribution and clearance processes within the device (Figure 3F,G) [50,51]. A drug introduced into the circulation compartment undergoes sequential exposure to metabolizing and target compartments, generating time-dependent concentration profiles that are shaped by both programmed input and tissue-mediated transformation [57,58].

A key conceptual distinction from earlier programmable systems lies in the emergence of PK dynamics from system-level interactions. Whereas previously described platforms reproduce predefined plasma curves, multi-compartment microfluidic systems allow concentration–time trajectories to arise from integrated transport, metabolism, and redistribution processes. The microfluidic device operates as a reduced-scale analog of a PBPK model, where compartmental volumes and flow fractions are engineered to preserve relative scaling relationships [60].

Experimental validation demonstrates that concentration profiles measured in downstream compartments reflect the combined influence of flow architecture and tissue-specific activity. For example, incorporation of a metabolically active liver module alters the temporal exposure experienced by a downstream tumor or target tissue chamber, resulting in delayed peaks or attenuated maximum concentrations compared with input conditions. Such behavior illustrates how organ-level interactions modulate effective PD exposure, even when initial dosing parameters are identical [58,60].

This architecture enables mechanistic interrogation of distribution-driven variability. By altering intercompartmental flow ratios or modifying the relative size of individual chambers, investigators can simulate conditions analogous to altered perfusion, impaired metabolism, or organ-specific accumulation. The resulting concentration–time patterns are not imposed externally but emerge from network-level fluidic interactions, providing a physical representation of compartmental PK [50,51].

These platforms also support quantitative comparison with computational PBPK predictions. Modeled human PK parameters can be translated into scaled microfluidic flow fractions and chamber volumes, after which experimentally measured concentration curves are compared against model outputs. Discrepancies between predicted and observed profiles inform refinement of scaling assumptions and kinetic parameters. In this manner, the microfluidic system becomes both an experimental execution tool and a validation environment for PBPK frameworks [55,60].

From a translational standpoint, integrating multi-tissue interaction within programmable microfluidic environments extends the scope of PK-informed in vitro modeling beyond isolated exposure. Drug concentration at the target tissue is no longer assumed to mirror plasma kinetics but is instead shaped by upstream metabolism and redistribution. This distinction becomes particularly relevant for compounds with extensive first-pass metabolism, active metabolites, or tissue-specific sequestration [30,58]. By capturing these layered dynamics within a controlled in vitro setting, multi-compartment microfluidic platforms move closer to reproducing physiologically meaningful exposure conditions [56].

Technically, implementation requires careful control of recirculation stability, minimization of dead volume, and verification of scaling relationships between compartments. Material adsorption and interfacial losses may disproportionately influence low-volume systems and must be characterized [51]. Nevertheless, by combining programmable dosing input with internally generated intercompartmental PK, these platforms represent a progression from curve replication toward dynamic system-level reconstruction of drug disposition [60].

## 3. PK–PD Coupling and Quantitative In Vitro–In Vivo Translation

Dynamic microfluidic platforms have enabled experimental evaluation of dosing strategies under physiologically relevant PK conditions, moving beyond static potency measurements toward schedule-dependent optimization [61,62]. A representative drug development case is the use of a microfluidic PK–PD platform to optimize dosing schedules for caseinolytic protease P (ClpP) agonists, a class of mitochondrial-targeting anticancer agents [61].

**Figure 4:**
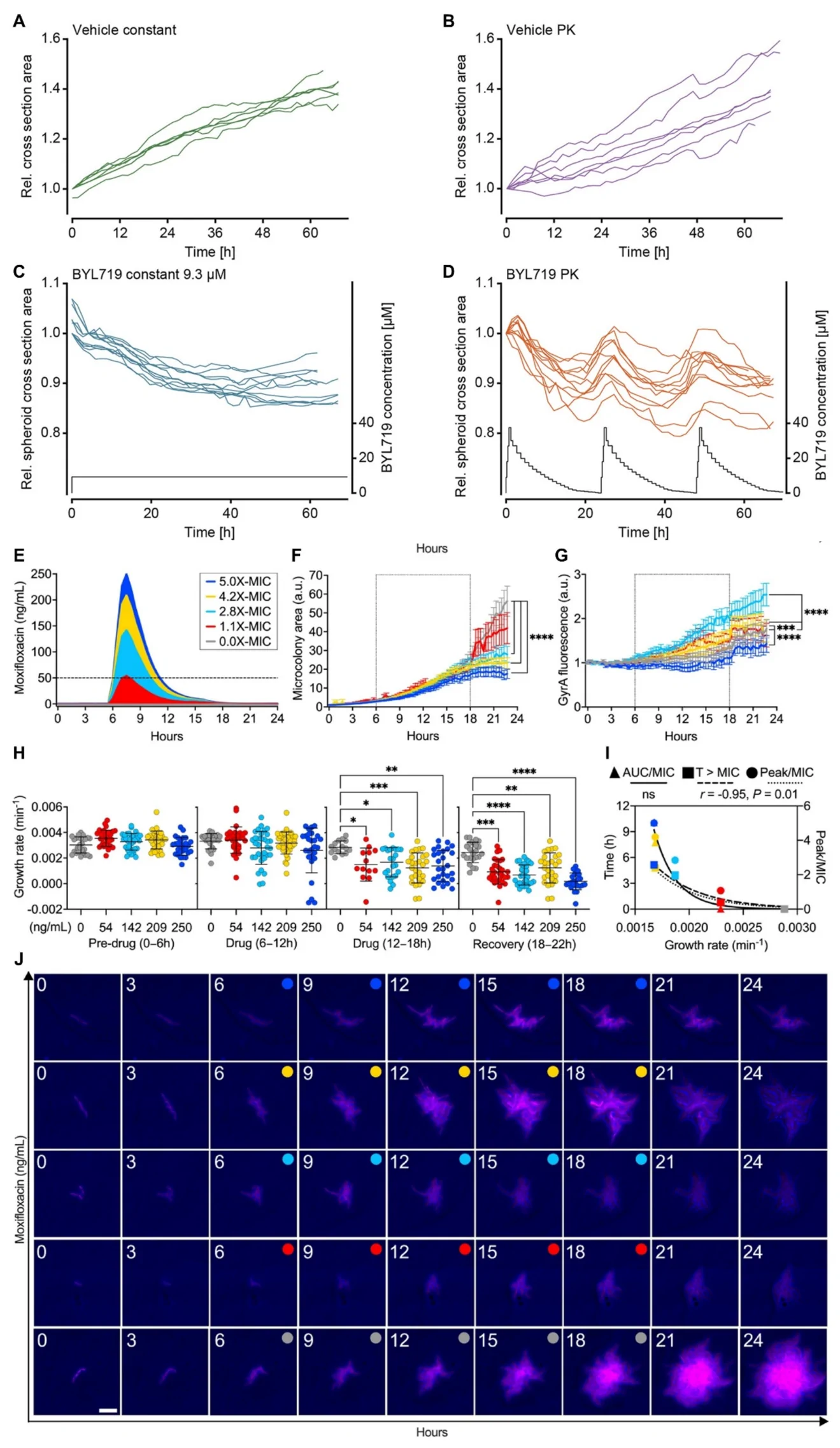
PK-dependent divergence of cellular response and quantitative PK–PD relationships under dynamic exposure conditions. (**A**,**B**) Time-dependent changes in spheroid growth under constant vehicle exposure (**A**) and vehicle PK-mimicking conditions (**B**), showing comparable baseline behavior. (**C**) Spheroid response under constant drug exposure (BYL719, 9.3 μM), resulting in sustained growth inhibition. (**D**) Dynamic PK exposure of BYL719, demonstrating a fluctuating spheroid response corresponding to time-varying concentration profiles (gray curve). (**E**) Representative PK profiles of moxifloxacin at varying exposure levels expressed as multiples of the minimum inhibitory concentration (MIC). (**F**) Quantification of microcolony area over time under different dynamic exposure conditions. (**G**) Fluorescence-based readout of bacterial response, indicating exposure-dependent activity dynamics. (**H**) Growth rate analysis across different exposure windows, highlighting temporal dependence of drug effects. (**I**) Correlation between PK metrics (AUC/MIC, T > MIC, and Peak/MIC) and growth rate, demonstrating that distinct PK parameters differentially predict biological response. (**J**) Time-lapse fluorescence imaging of bacterial microcolonies exposed to different moxifloxacin concentrations, illustrating spatial and temporal heterogeneity in growth dynamics. (* *p* < 0.05, ** *p* < 0.01, *** *p* < 0.001, and **** *p* < 0.0001.) Reproduced under terms of the CC-BY license. References [10,68], Copyright 2021, The Authors, published by Frontiers Media S.A.; Copyright 2022, The Authors, published by Springer Nature.

In this study, experimentally derived PK profiles were reproduced within a microfluidic device to assess how different dosing regimens influence PD outcomes. Instead of maintaining constant drug concentrations, the platform generated time-varying exposure curves that reflected clinically relevant peak levels, elimination rates, and dosing intervals [61]. By comparing bolus-like high-peak exposures with prolonged lower-concentration regimens under controlled flow conditions, the investigators demonstrated that cellular response was highly sensitive to the temporal structure of exposure (Figure 4A–D).

The study revealed that equivalent area under the concentration–time curve did not necessarily translate into equivalent biological effects [63]. Regimens producing higher peak concentrations over shorter durations induced distinct mitochondrial stress responses and growth inhibition patterns compared with regimens delivering the same total exposure more gradually. This divergence underscored the inadequacy of static IC_50_-based evaluation for compounds whose mechanism of action depends on transient mitochondrial disruption and recovery kinetics [63].

The microfluidic platform enabled systematic manipulation of dosing frequency and interval while maintaining reproducible concentration trajectories. By altering the timing between successive exposure pulses, the investigators observed nonlinear accumulation of PD effects, suggesting that cellular recovery dynamics modulate sensitivity to repeated treatment. These findings highlight that optimal therapeutic scheduling cannot be inferred solely from static dose–response curves but requires explicit incorporation of time-dependent PK information [64,65].

Translationally, the study illustrates how microfluidic PK replication can serve as an intermediate testing environment between computational modeling and in vivo experimentation. Hypothesized dosing regimens, derived from preclinical or clinical PK parameters, can be implemented directly within the device and evaluated for mechanistic response patterns [62]. This approach reduces reliance on empirical trial-and-error dose escalation in animal models and allows more rational identification of regimens that maximize PD impact under realistic exposure constraints [62].

By experimentally demonstrating that dosing schedule, peak concentration, and temporal spacing independently shape drug efficacy, the ClpP agonist study provides concrete evidence that dynamic PK–PD coupling is essential for accurate in vitro evaluation [64,65]. Rather than treating concentration as a static variable, the system frames drug action as a function of exposure history, thereby laying the groundwork for more quantitative integration of PK and PD within microphysiological platforms [66,67].

Building upon schedule-dependent dose optimization, microfluidic platforms have further enabled the redefinition of dose–response relationships under dynamically controlled PK conditions [68]. Traditional dose–response analysis assumes constant exposure and derives potency metrics such as IC_50_ from equilibrium conditions. However, when drug concentration varies over time, the concept of “dose” itself becomes multidimensional, incorporating not only magnitude but also duration, rate of change, and temporal sequencing [64,65].

The microfluidic dose–response framework addresses this limitation by generating controlled, time-varying exposure profiles while systematically modulating concentration parameters [69]. Rather than testing a series of fixed concentrations, the system produces dynamic concentration trajectories that differ in peak amplitude, exposure duration, or frequency of administration. Cellular responses are then mapped against these structured exposure histories, enabling construction of dynamic dose–response landscapes rather than single static curves [68].

A central finding of this approach is that dose–response relationships are not invariant across exposure schedules. Identical nominal concentrations may yield divergent outcomes depending on whether they are delivered as a short high-concentration pulse or a prolonged lower-intensity exposure. In some cases, transient exposure above a critical threshold triggers irreversible PD effects, whereas sustained subthreshold exposure produces minimal response despite comparable cumulative AUC [68]. These observations challenge the conventional assumption that potency can be summarized by a single concentration metric independent of time [64,65].

The microfluidic system enables decoupling of exposure variables that are confounded in static assays. Peak concentration (C_max_), time above a pharmacologically active threshold, and rate of concentration decline can be independently varied while holding other parameters constant [70]. By analyzing response surfaces across these orthogonal exposure dimensions, investigators can identify nonlinearities and tipping points in PD behavior [66,71]. This multidimensional mapping reveals that cellular sensitivity is often governed by exposure history rather than by instantaneous concentration alone [68].

The study also demonstrates that dynamic dose–response relationships may reshape interpretation of the therapeutic index [66]. Regimens that appear equivalent under static IC_50_ evaluation can diverge substantially when temporal dynamics are introduced. Certain exposure patterns may preferentially amplify cytotoxic effects while minimizing recovery, whereas others allow adaptive or compensatory responses to emerge. Thus, dose–response analysis in a dynamic framework shifts emphasis from static potency ranking toward temporal optimization [64,65].

From a quantitative perspective, dynamic dose–response platforms facilitate integration of time-resolved exposure data into PD modeling [66]. By fitting experimental outcomes to models incorporating concentration–time input functions, parameters such as activation thresholds, response delay constants, and saturation kinetics can be estimated under physiologically relevant exposure conditions [72]. This modeling integration supports more accurate prediction of in vivo behavior compared with static potency extrapolation [66].

Collectively, microfluidic dose–response systems extend the principle established in schedule-dependent optimization studies by formalizing the relationship between time-varying PK and PD effects [61]. Rather than treating concentration as a static scalar variable, these platforms conceptualize dose as a dynamic trajectory, thereby providing a quantitative bridge toward more mechanistically grounded PK–PD coupling [67].

While dynamic dose–response studies demonstrate that temporal exposure structure reshapes PD outcomes, a more systematic framework is required to quantitatively formalize this relationship. The concept of micro-PK–PD extends beyond empirical schedule comparison by explicitly integrating time-resolved concentration data with mechanistic PD modeling [14,73]. In this framework, drug concentration is treated as a continuously evolving input variable that drives downstream biological processes, rather than as a static scalar parameter [70,74].

Micro-PK–PD platforms combine programmable microfluidic exposure control with synchronized measurement of cellular response, enabling direct correlation between concentration–time trajectories and PD endpoints. Unlike conventional assays that approximate exposure using nominal medium concentration, these systems capture the exact temporal profile experienced by cells. This distinction is critical when response dynamics involve lag phases, threshold effects, or recovery kinetics that depend on exposure history [67,70].

A defining feature of the micro-PK–PD approach is its capacity to disentangle exposure metrics that are otherwise conflated in static experiments [67]. Instead of relying solely on integrated measures, such as AUC, or single-point metrics, such as C_max_, the framework evaluates how full concentration–time waveforms influence biological effect (Figure 4E–H) [70]. Time above a pharmacologically active threshold, rate of concentration change, and temporal spacing between exposure pulses can be incorporated into quantitative models describing target engagement and downstream signaling cascades [74].

By embedding differential equation-based PD models within experimentally controlled microfluidic systems, micro-PK–PD platforms allow estimation of parameters governing sensitivity, maximal response, and delay constants under realistic kinetic conditions [72]. This integration enables testing of whether PD behavior aligns with classical maximum-effect (E_max_) models or sigmoid models when exposure is time-varying, or whether alternative mechanistic formulations are required [74]. As a result, PK–PD coupling becomes an experimentally verifiable construct rather than a purely theoretical extrapolation [14,67].

The micro-PK–PD paradigm provides a structured pathway toward in vitro–in vivo translation [66]. Instead of extrapolating static IC_50_ values to systemic plasma concentrations, experimentally measured dynamic exposure–response relationships can be mapped directly onto modeled or clinically observed PK curves [67]. This mapping enables evaluation of whether exposure windows achievable in patients correspond to concentration–time regions associated with maximal PD effect in vitro. In doing so, the system narrows the translational gap by aligning experimental exposure conditions with physiologically relevant kinetics [61].

Beyond temporal integration, micro-PK–PD analysis can incorporate spatial heterogeneity within microphysiological constructs. In three-dimensional cellular assemblies or perfused tissue models, diffusion limitations and local metabolic activity create gradients in drug concentration (Figure 4J) [75]. Coupling spatially resolved concentration measurement with dynamic exposure control permits construction of localized exposure–response relationships, further refining predictive accuracy [76,77].

Through quantitative coupling of dynamic PK and mechanistic PD, micro-PK–PD platforms move in vitro experimentation toward a model-informed framework. Rather than treating exposure and response as separate domains, the approach unifies them within a controlled microenvironment, establishing a basis for more predictive and physiologically grounded drug evaluation [67].

Extending quantitative micro-PK–PD integration into three-dimensional cellular systems, spatially resolved spheroid models provide an additional layer of translational relevance by incorporating tissue-like architecture and intratumoral heterogeneity [78]. Unlike monolayer cultures, multicellular spheroids develop diffusion gradients for oxygen, nutrients, and drugs, resulting in spatially heterogeneous exposure profiles that more closely resemble in vivo tumor microenvironments [73]. Within this context, PK variability is no longer solely temporal but also spatial [75,77].

In the spatial stable isotope labeling by amino acids in cell culture (SILAC)-based spheroid platform, chemotherapeutic exposure is combined with mass-spectrometry-enabled quantitative proteomic analysis to interrogate how drug concentration gradients translate into region-specific PD responses [79]. By applying stable isotope labeling and spatially resolved sampling, the system enables mapping of molecular alterations across different zones of the spheroid following defined exposure conditions. This approach captures not only overall cytotoxicity but also differential activation of stress pathways, apoptosis signaling, and metabolic adaptation as a function of spatial drug penetration [77].

The study demonstrates that nominal external drug concentration does not uniformly determine the intracellular PD effect throughout the spheroid structure. Peripheral cell layers, directly exposed to circulating medium, experience higher effective concentrations compared with hypoxic or diffusion-limited core regions [77]. As a consequence, identical global dosing conditions generate heterogeneous PK–PD coupling within the same microtissue [80]. This spatial divergence may partially explain discrepancies between in vitro potency measurements and in vivo tumor response, where drug distribution is rarely uniform [73].

By integrating time-resolved exposure control with spatial proteomic quantification, the platform refines the micro-PK–PD paradigm beyond bulk measurements [77]. PD outcomes can be interpreted as a composite of localized exposure histories, reflecting both temporal concentration changes and radial diffusion dynamics. This dual resolution provides insight into threshold-dependent responses that may occur only in specific microregions, as well as adaptive signaling mechanisms that emerge under sublethal exposure conditions in the tumor core [81].

From a translational standpoint, spatially resolved PK–PD analysis addresses a critical limitation of conventional assays: the assumption of homogeneous drug distribution [76]. In vivo, tumor perfusion variability, stromal barriers, and interstitial pressure gradients generate complex exposure landscapes [80]. By reproducing analogous gradients in vitro and quantitatively linking them to molecular response patterns, spheroid-based PK–PD systems offer a more physiologically grounded evaluation of chemotherapeutic efficacy [79].

Technically, successful implementation requires precise coordination between controlled exposure conditions and downstream spatial analytics. Drug diffusion kinetics, spheroid size, and sampling resolution influence the accuracy of spatial mapping [75]. Nonetheless, the integration of dynamic PK with spatially resolved PD represents a significant conceptual expansion of micro-PK–PD methodology. Rather than evaluating exposure–response relationships at a single averaged concentration, the system reconstructs multidimensional PK–PD interactions across time and space, thereby advancing the predictive capacity of in vitro microphysiological platforms [79,80].

As a representative drug development case, the ClpP agonist study illustrates how dynamic MPSs can support dosing-regimen selection by linking exposure timing, PD response, and in silico PK–PD prediction, thereby translating microfluidic exposure control into a practical scheduling decision.

## 4. Spatial and Microenvironmental PK

Beyond temporal PK control, spatial heterogeneity within tissues strongly influences PD outcomes [82]. In solid tumors and other dense microtissues, drug distribution is rarely uniform due to diffusion barriers, heterogeneous perfusion, and variable cellular uptake [83]. As a result, cells located at different spatial positions within the same tissue construct may experience markedly different effective drug concentrations, even under identical systemic dosing conditions. Capturing spatial PK is essential for realistic exposure–response analysis [84].

**Figure 5:**
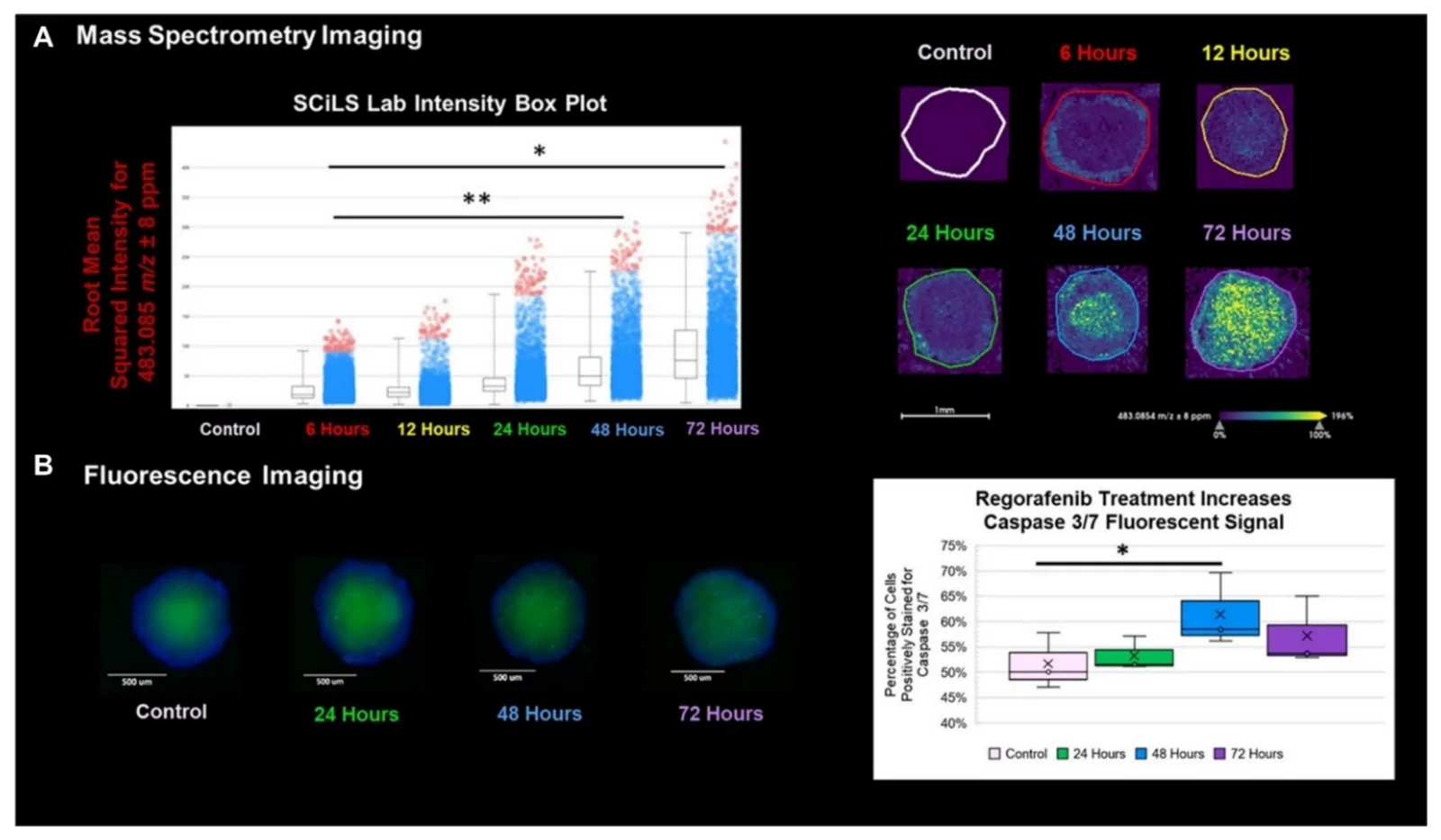
Spatially resolved drug distribution and corresponding pharmacodynamic responses in 3D microtissues. (**A**) Mass spectrometry imaging (MSI) of drug distribution within spheroids over time, with quantitative intensity analysis (left) and representative spatial maps (right) showing progressive intratissue penetration. (**B**) Fluorescence imaging and caspase-3/7 quantification showing corresponding spatial and temporal pharmacodynamic responses after treatment. (* *p* < 0.05 and ** *p* < 0.01.) Reproduced under terms of the ACS AuthorChoice license. Reference [77], Copyright 2023, American Chemical Society.

Matrix-assisted laser desorption/ionization (MALDI) imaging mass spectrometry has emerged as a powerful tool for visualizing drug distribution within three-dimensional cellular assemblies [85,86]. By enabling spatial detection of molecules within tissue sections, MALDI imaging provides quantitative maps of intratissue drug penetration (Figure 5A). When applied to multicellular spheroids or tumor models, this approach reveals concentration gradients extending from peripheral layers toward hypoxic or necrotic cores. These gradients often correlate with biological responses not captured by bulk measurements (Figure 5B) [81].

**Figure 6:**
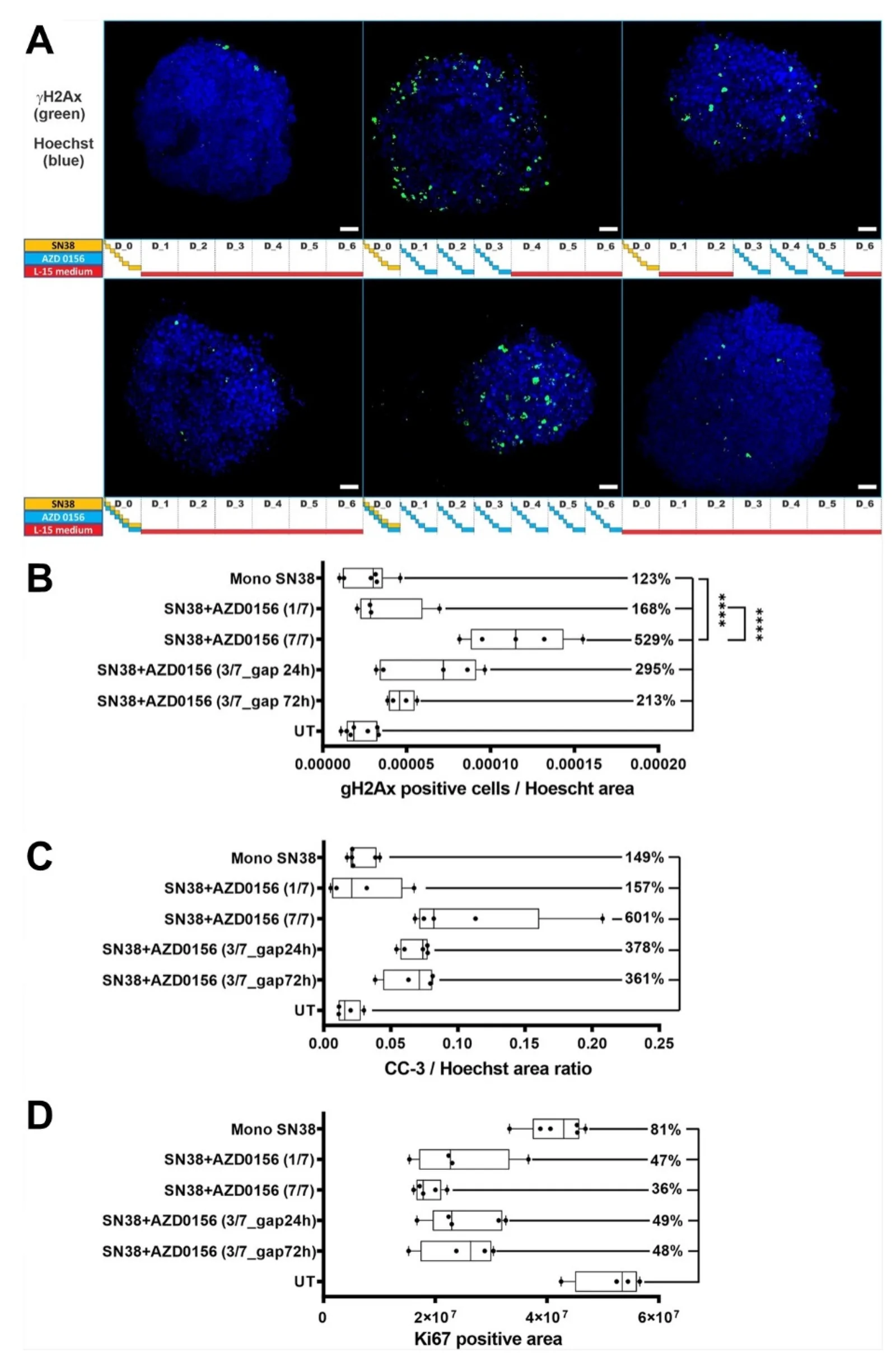
Schedule-dependent spatial pharmacodynamic responses in 3D tumor spheroids under dynamic drug exposure. (**A**) Representative fluorescence images of spheroids stained for phosphorylated histone H2AX (γH2AX; green) and Hoechst (blue) under different treatment schedules of SN38 and AZD0156, showing spatially heterogeneous DNA damage responses. (**B**) Quantification of DNA damage based on γH2AX-positive cells normalized to Hoechst area across treatment conditions. (**C**) Apoptotic response quantified by cleaved caspase-3 (CC-3) signal, demonstrating schedule-dependent variation in cell death. (**D**) Proliferation analysis based on Ki67-positive area, indicating differential effects of treatment timing and sequence on tumor cell growth. (**** *p* < 0.0001.) Reproduced under terms of the CC-BY license. Reference [91], Copyright 2021, The Authors, published by Communications Biology.

The integration of MALDI imaging with spatial SILAC-based quantitative proteomics further extends this framework by linking spatial PK to region-specific PD signaling. Stable isotope labeling enables identification of spatial proteomic changes across spheroid regions after drug exposure (Figure 6A) [85]. This approach reveals both drug accumulation and local molecular responses. Consequently, PD heterogeneity reflects spatial exposure variability rather than biological noise (Figure 6B) [87].

Importantly, spatially resolved analyses demonstrate that nominal extracellular drug concentration does not uniformly determine intracellular PD response across a three-dimensional structure [85]. Peripheral cells exposed to higher concentrations may undergo rapid apoptosis or stress responses, whereas cells in diffusion-limited core regions experience sublethal exposure that permits adaptive metabolic reprogramming (Figure 6C,D) [83]. Distinct PK–PD states within a single microtissue challenge predictions based on averaged exposure metrics [82].

From a translational standpoint, spatial PK characterization addresses a critical limitation of conventional in vitro assays, which assume homogeneous drug distribution. In vivo tumors exhibit complex perfusion patterns, stromal barriers, and interstitial pressure gradients that create heterogeneous exposure landscapes [84]. By reconstructing such gradients in controlled three-dimensional systems, MALDI imaging combined with spatial proteomics provides mechanistic insight into penetration-limited efficacy and resistance phenomena [85].

Technically, accurate spatial PK–PD mapping requires careful calibration of sectioning depth, ionization efficiency, and normalization strategies to ensure quantitative reliability [86,88]. Drug diffusion kinetics, spheroid size, and extracellular matrix composition all influence gradient formation and must be considered when interpreting results [83]. Nevertheless, by integrating molecular imaging with spatially resolved PD profiling, this approach advances microphysiological modeling beyond temporal exposure control toward multidimensional reconstruction of drug behavior within complex tissue architectures [85].

While spatial imaging and proteomic mapping reveal heterogeneous drug penetration within static three-dimensional constructs, tumor-on-chip microfluidic platforms introduce controlled perfusion and vascular mimicry to further approximate in vivo microenvironments [89]. In contrast to non-perfused spheroids, tumor-on-chip systems incorporate microfluidic channels that simulate vascular flow, enabling dynamic delivery of therapeutics under defined shear stress and flow-rate conditions [90]. This configuration allows simultaneous control of temporal PK input and spatial transport processes [91].

A defining advantage of tumor-on-chip platforms lies in their ability to model the interplay between perfusion, interstitial transport, and cellular uptake. Drug molecules delivered through perfusable microchannels diffuse across extracellular matrix regions and penetrate tumor-like cellular compartments, generating gradients shaped by flow dynamics and tissue architecture [91]. Unlike static diffusion-limited systems, these devices allow systematic manipulation of flow rate, channel geometry, and matrix composition to examine how transport barriers influence effective exposure at the cellular level [92].

Tumor-on-chip studies demonstrate that microvascular flow patterns significantly modulate intratumoral drug distribution [91]. Regions located proximal to perfusion channels receive higher effective concentrations, whereas distal or poorly perfused zones exhibit delayed or attenuated exposure [93]. These spatial PK differences produce heterogeneous PD responses, including variable apoptosis induction and proliferation inhibition across the construct. These findings show that effective exposure depends on both dose and transport accessibility [92].

The integration of controlled flow with real-time imaging further enables quantification of penetration kinetics. Time-resolved measurements of fluorescently labeled compounds or surrogate tracers allow reconstruction of concentration gradients as they evolve under perfusion conditions [93,94]. By correlating these spatial PK measurements with downstream biological readouts, tumor-on-chip systems provide a mechanistic framework for understanding how vascular architecture and microenvironmental resistance influence therapeutic efficacy [95].

From a translational perspective, perfused tumor-on-chip platforms address limitations inherent to both static in vitro assays and oversimplified PK modeling. In vivo tumors are characterized by abnormal vasculature, heterogeneous perfusion, and elevated interstitial pressure, all of which shape drug delivery [96]. By recapitulating these features within a controllable microfluidic environment, tumor-on-chip systems enable systematic evaluation of transport-limited exposure scenarios that may underlie treatment failure despite adequate systemic plasma levels [94].

Technically, accurate modeling requires careful calibration of flow scaling, matrix permeability, and channel dimensions to ensure physiologically relevant transport regimes [92]. Material adsorption, pressure stability, and long-term culture viability must also be validated to maintain consistent exposure conditions [91]. Nevertheless, by integrating vascularized perfusion with three-dimensional tumor architecture, tumor-on-chip platforms extend spatial PK analysis from passive diffusion gradients toward dynamic microenvironmental reconstruction [94].

Together, MALDI imaging, spatial proteomics, and tumor-on-chip models reveal multidimensional PK, governed by both temporal dosing structure and spatial transport constraints [85]. Addressing this dual complexity is essential for quantitative in vitro–in vivo translation in solid tumors and other heterogeneous tissues [82].

## 5. From Single-Organ PK to Multi-Organ Human Prediction

### 5.1. Intestinal Absorption and Barrier Models

Systemic PK prediction requires accurate reconstruction of absorption processes at biological barriers [97]. Among these, the intestinal epithelium represents a primary determinant of oral drug exposure, governing systemic drug entry. Traditional static Transwell-based permeability assays provide apparent permeability coefficients under equilibrium-like conditions, yet they fail to capture dynamic concentration gradients, luminal flow, and time-dependent absorption kinetics that characterize in vivo intestinal transport [98,99].

Dynamic in vitro intestinal barrier models address these limitations by incorporating controlled perfusion across epithelial monolayers cultured within microfluidic architectures [100]. In these systems, apical and basolateral compartments are independently perfused, allowing modulation of luminal drug concentration while quantifying transepithelial flux over time [101]. By introducing defined flow rates, shear stress, and concentration decay profiles, the platform reproduces non-equilibrium transport conditions more closely aligned with physiological absorption dynamics [102].

A key feature of the dynamic intestinal barrier model lies in its ability to generate time-resolved permeability profiles rather than single steady-state permeability values [100]. Instead of reporting a static apparent permeability coefficient (Papp), the system captures evolving transport rates as luminal concentration declines or fluctuates [101]. This temporal resolution enables discrimination between passive diffusion, carrier-mediated uptake, and efflux transporter activity under realistic exposure histories [98]. Thus, absorption kinetics depend on both concentration and exposure duration.

Importantly, coupling dynamic exposure control with quantitative sampling allows integration of experimentally measured transport data into PK modeling frameworks [103]. Measured flux across the epithelial barrier can be translated into effective absorption rate constants, which may then be incorporated into compartmental or PBPK models. This integration provides a mechanistic bridge between microscale transport measurements and systemic plasma concentration prediction [101].

The dynamic intestinal barrier approach also permits investigation of first-pass processes, including transporter saturation, metabolic conversion within epithelial cells, and time-dependent permeability changes [98]. Because concentration–time profiles can be programmed to mimic clinical dosing scenarios, the model enables evaluation of how rapidly changing luminal concentrations influence net absorption efficiency [102]. Such analysis is particularly relevant for compounds exhibiting nonlinear uptake or significant efflux modulation [98].

Translationally, dynamic intestinal models move beyond ranking compounds by static permeability and toward reconstructing absorption-driven systemic PK [97]. By embedding barrier transport within a controlled and time-resolved microfluidic environment, these systems contribute a critical upstream component to multi-organ PK reconstruction [19]. Accurate modeling of intestinal absorption provides the foundation for integrating hepatic metabolism and systemic distribution modules [104,105].

While dynamic intestinal barrier models improve temporal control of absorption kinetics, organotypic intestinal chips further enhance physiological relevance by recapitulating the structural and functional complexity of the human small intestine [106]. The Human Duodenum Chip integrates primary intestinal epithelial cells within a perfused microfluidic device [107]. This configuration supports differentiated intestinal epithelium under flow.

**Figure 7:**
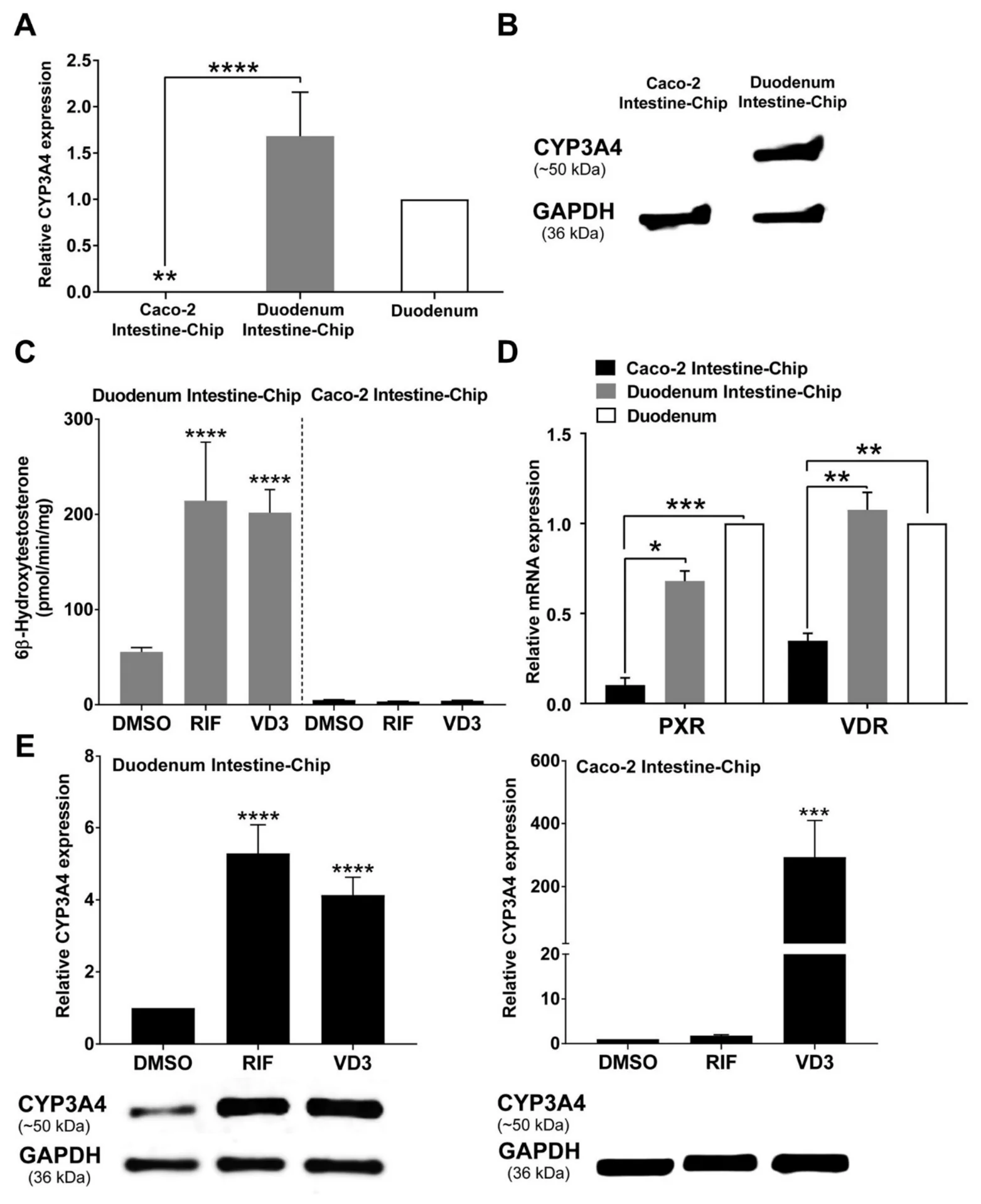
Functional metabolic competence of organotypic intestinal chip models. (**A**) Relative CYP3A4 expression in the Caco-2 intestine-chip and human duodenum intestine-chip compared with native tissue. (**B**) Representative Western blot analysis confirming CYP3A4 protein expression in different intestinal models. (**C**) Induction of CYP3A4 expression in the duodenum intestine-chip and Caco-2 intestine-chip following treatment with rifampicin (RIF) and vitamin D3 (VD3). (**D**) Functional metabolic activity assessed by 6β-hydroxytestosterone production, demonstrating active CYP3A4-mediated metabolism in the duodenum intestine-chip. (**E**) Relative mRNA expression of nuclear receptors pregnane X receptor (PXR) and vitamin D receptor (VDR) across intestinal models, supporting regulatory control of drug-metabolizing enzymes. (* *p* < 0.05, ** *p* < 0.01, *** *p* < 0.001, and **** *p* < 0.0001.) Reproduced under terms of the CC-BY license. Reference [107], Copyright 2020, The Authors, published by eLife.

A key advancement of the Human Duodenum Chip lies in its ability to reproduce transporter expression profiles and metabolic enzyme activity comparable to those observed in native human tissue [108]. Unlike conventional cell lines that often exhibit altered or diminished expression of cytochrome P450 enzymes and efflux transporters, the chip platform preserves functional levels of cytochrome P450 3A4 (CYP3A4) and other drug-metabolizing enzymes (Figure 7A,B) [107]. This feature allows simultaneous assessment of absorption and first-pass metabolism within a controlled in vitro system (Figure 7C,D) [108].

Under perfused conditions, luminal drug exposure can be dynamically modulated, while basolateral sampling quantifies absorbed and metabolized fractions over time. The integration of mechanical stretch further mimics peristalsis-like motion, influencing epithelial morphology and functional maturation [106]. As a result, transport and metabolic kinetics measured within the Duodenum Chip reflect more physiologically representative behavior compared with static monolayer cultures [107].

The system supports evaluation of drug–drug interactions and transporter-mediated effects under clinically relevant concentration–time profiles. Induction or inhibition of metabolic enzymes can be assessed in a time-resolved manner, enabling mechanistic characterization of compounds whose systemic exposure depends on intestinal metabolism (Figure 7E) [108]. Such functionality strengthens the predictive potential of the model when incorporated into broader PK reconstruction frameworks [103].

As a practical absorption and DDI case example, the Human Duodenum Chip demonstrates how organotypic intestinal MPSs can be used to evaluate transporter expression, CYP3A4-mediated metabolism, and inducible drug-metabolizing activity under perfused conditions [107].

From a systems perspective, the Human Duodenum Chip represents a transitional stage between isolated barrier transport models and fully integrated multi-organ platforms [107]. By accurately capturing intestinal absorption and metabolic transformation under dynamic conditions, the model provides quantitatively reliable input parameters for downstream hepatic and systemic modules [104]. Organotypic intestinal chips contribute not merely qualitative validation but mechanistic data suitable for integration into PBPK modeling [108].

Technically, maintenance of differentiated epithelial phenotypes requires careful control of flow rate, oxygenation, and extracellular matrix composition. Long-term stability and reproducibility across donors remain critical considerations for translational application [106]. Nevertheless, by combining microfluidic perfusion, human-derived cellular architecture, and functional metabolic competence, the Duodenum Chip advances in vitro intestinal modeling from permeability estimation toward system-level PK relevance.

While organotypic intestinal chips improve the physiological fidelity of absorption modeling, systemic PK following oral administration is critically shaped by coordinated intestine–liver interactions [109]. After transepithelial transport, absorbed compounds enter the portal circulation and undergo hepatic metabolism before reaching systemic distribution [105]. Reconstructing first-pass metabolism in vitro requires integration of intestinal and hepatic modules [110].

**Figure 8:**
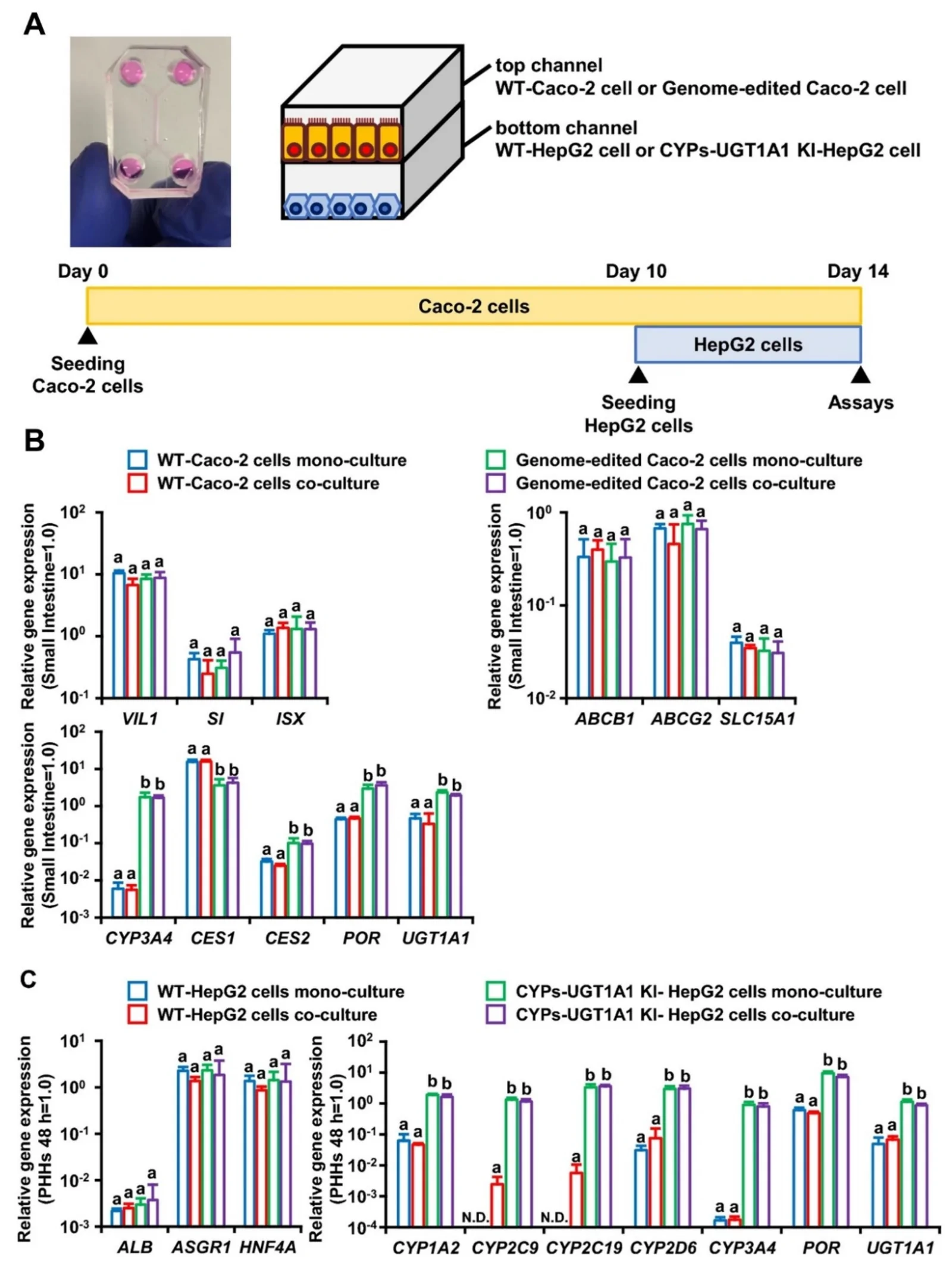
Integrated intestine–liver microphysiological system with tunable metabolic capacity. (**A**) Schematic of the coupled intestine–liver chip platform, including epithelial (Caco-2 or genome-edited Caco-2) and hepatic (HepG2 or CYPs–UGT1A1 knock-in HepG2) compartments, along with experimental timeline for cell seeding and co-culture. (**B**) Gene expression profiles of intestinal epithelial markers and transporters in mono- and co-culture conditions, demonstrating maintenance of intestinal phenotype and functional transport properties. (**C**) Hepatic gene expression analysis of metabolic enzymes in mono- and co-culture systems, highlighting modulation of cytochrome P450 and UGT pathways through genome editing and inter-organ interaction. (Groups that do not share the same letter (a, b) are significantly different (*p* < 0.05). N.D., not detected.) Reproduced under terms of the CC-BY license. Reference [110], Copyright 2023, The Authors, published by Scientific Reports.

Genome-edited intestine–liver platforms address this challenge by combining differentiated intestinal epithelium with metabolically competent liver tissue in interconnected microfluidic systems (Figure 8A) [110]. In certain implementations, targeted genome editing is employed to modulate specific metabolic enzymes or transporters, enabling controlled investigation of their contribution to systemic exposure. By engineering defined expression profiles within intestinal or hepatic compartments, the model permits mechanistic dissection of how individual pathways influence overall PK behavior (Figure 8B) [105].

In these coupled systems, a drug introduced into the luminal compartment first traverses the intestinal barrier under perfusion conditions, after which the absorbed fraction is routed to a downstream hepatic chamber [109]. The liver module metabolizes parent compounds into measurable metabolites [58]. This sequential configuration reconstructs portal-vein-like transfer and allows dynamic measurement of first-pass extraction ratios under programmable concentration–time inputs [110].

A major advantage of the intestine–liver integration lies in its ability to capture nonlinear or saturation-dependent metabolism arising from coordinated transporter and enzyme activity [105]. Because concentration–time profiles can be dynamically controlled at the intestinal interface, investigators can examine how variations in luminal exposure propagate through hepatic clearance processes [104]. This is particularly relevant for compounds exhibiting transporter–enzyme interplay or time-dependent metabolic induction [110].

Furthermore, genome editing enables simulation of genetic variability associated with altered metabolic capacity [110]. By selectively knocking down or overexpressing key enzymes, such as CYP isoforms, the platform can model interindividual differences in clearance and systemic exposure (Figure 8C). Such capability provides an experimental complement to in silico sensitivity analyses commonly performed in PBPK modeling [103].

From a translational standpoint, the intestine–liver microphysiological axis represents a critical intermediate step toward predictive human PK [19,104]. By experimentally reconstructing absorption and first-pass metabolism within a controlled and modular environment, these systems generate quantitative parameters that can inform PBPK models [109]. Rather than estimating first-pass extraction solely from isolated hepatic microsomes or static permeability data, integrated platforms enable direct measurement of systemic availability under dynamic and physiologically structured exposure conditions.

Technically, successful operation requires stable recirculation, physiologically scaled flow ratios between intestinal and hepatic compartments, and careful characterization of compound adsorption within the microfluidic network [109]. Calibration of residence time and volume scaling is essential to maintain proportional relationships analogous to human physiology [58]. Nonetheless, by linking genome-level modulation with organ-level interaction, intestine–liver platforms extend microphysiological modeling from barrier characterization toward systemic first-pass PK reconstruction [60].

### 5.2. Multi-Tissue and Vascularized Systems

**Figure 9:**
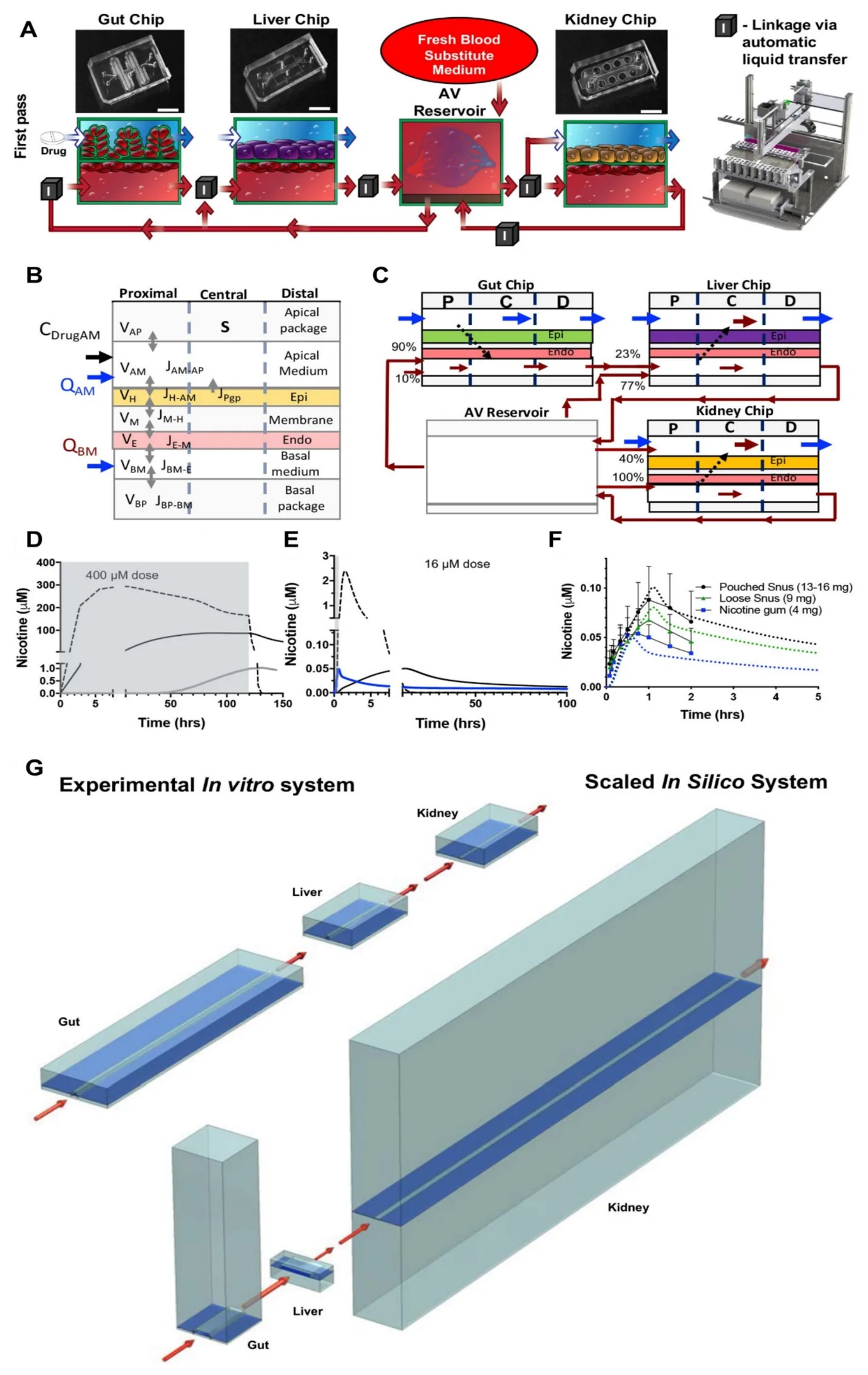
Multi-organ microphysiological system for systemic PK reconstruction and in vitro–in silico integration. (**A**) Schematic of a vascularized multi-organ platform integrating gut, liver, and kidney chips connected through a recirculating medium reservoir, modeling absorption, metabolism, and clearance. (**B**) Compartmental representation of transport processes across epithelial and endothelial barriers, illustrating apical–basolateral exchange and intercompartmental fluxes. (**C**) Integrated flow network linking organ modules, highlighting distribution of drug fractions and inter-organ transport dynamics under perfused conditions. (**D**–**F**) Representative concentration–time profiles demonstrating dose-dependent PK behavior and comparison with clinically relevant exposure patterns. (**G**) Conceptual framework linking experimental multi-organ MPSs with scaled in silico PBPK models, enabling quantitative prediction of systemic drug disposition. Reproduced with permission from Ref. [118]. Copyright 2020, Springer Nature.

Multi-tissue microphysiological platforms extend intestine–liver models to reconstruct systemic PK through integrated organ interactions [111]. Rather than focusing solely on absorption or first-pass metabolism, these systems incorporate multiple functional tissue compartments—commonly liver, kidney, and additional peripheral tissues—within a recirculating microfluidic network (Figure 9A) [112]. In this configuration, drug disposition emerges from coordinated metabolic transformation, renal clearance, and tissue distribution processes that collectively approximate whole-body PK [112].

In the multi-tissue chip platform integrating liver, kidney, and skeletal muscle modules, individual organ compartments are fluidically interconnected under defined scaling relationships that reflect physiological volume fractions and flow distributions (Figure 9B). A drug introduced into the circulation undergoes hepatic metabolism, renal clearance, and peripheral tissue uptake (Figure 9C). Over time, concentration–time profiles measured within the recirculating system reflect composite clearance processes rather than externally imposed concentration waveforms (Figure 9D–F) [113].

A key objective is quantitative prediction of human PK parameters, such as clearance (CL), half-life (t_1_/_2_), and volume of distribution (V_d_) [114]. By measuring time-resolved drug concentrations within the circulating compartment and applying appropriate scaling factors, investigators derive intrinsic clearance values that can be extrapolated to human systemic parameters [115]. This approach moves beyond qualitative assessment of metabolic activity and toward numerical estimation of clinically relevant PK metrics [112].

The inclusion of a metabolically competent liver module enables characterization of hepatic intrinsic clearance under dynamic flow conditions, while the kidney compartment contributes active secretion or filtration components that influence the overall elimination rate [116]. Peripheral tissues act as distribution sinks shaping the apparent volume of distribution and elimination kinetics. The interplay among these compartments generates concentration–time trajectories that approximate multi-compartment PK behavior observed in vivo [113].

Crucially, the system is not limited to reproducing predefined plasma curves but instead allows PK dynamics to arise from integrated organ-level processes [111]. Clearance emerges as a function of intrinsic metabolic capacity, intercompartmental flow, and tissue binding within the microphysiological environment. Thus, concentration decline reflects internally generated system behavior rather than programmed decay [117].

To translate chip-derived measurements into human predictions, scaling frameworks are applied to account for relative organ mass, flow fraction, and cellular metabolic capacity (Figure 9G) [117]. Intrinsic clearance from the liver module is normalized to cell number and extrapolated to whole-organ scale using in vitro–in vivo extrapolation (IVIVE) principles. Similarly, renal clearance components are adjusted based on physiological filtration rates and transporter activity assumptions [116]. Resulting clearance estimates are compared with clinical PK data to assess predictive accuracy [114].

Validation studies demonstrate that multi-tissue chip platforms can approximate human PK parameters within a reasonable fold-error range for selected compounds. Predictive performance improves when organ scaling and flow ratios are calibrated using PBPK modeling frameworks [118]. In this context, the microphysiological system functions as an experimental implementation of PBPK structure, providing empirical parameter inputs rather than relying solely on literature-derived estimates [112].

From a translational perspective, multi-tissue PK platforms represent a shift from exposure emulation toward systemic disposition reconstruction [111]. Rather than prescribing concentration–time inputs, these systems generate PK outputs through coordinated organ interaction. This is critical for drugs whose systemic behavior depends on competing metabolic and distribution pathways [113].

Technically, accurate prediction requires careful control of recirculation stability, minimization of dead volume, and validation of compound adsorption within device materials [119]. Precise scaling of intercompartmental flow fractions is essential to preserve physiologically meaningful clearance contributions [115]. Despite these challenges, the integration of multiple human-relevant tissues within a unified microfluidic network establishes a quantitative framework for predicting systemic PK directly from in vitro MPSs [112].

Beyond multi-tissue clearance reconstruction, vascularized organ-chip platforms introduce a higher level of physiological integration by incorporating endothelial-lined microvascular networks that enable systemic recirculation across interconnected human tissue modules [59]. In these systems, drug distribution is not governed solely by programmed flow between compartments but by perfusion through tissue-specific vascular interfaces that more closely approximate in vivo hemodynamic architecture [118].

In the vascularized multi-organ platform integrating liver, intestine, kidney, and additional tissue modules, each organ unit is connected through a common microfluidic circulation channel lined with human endothelial cells [59]. This vascular layer mediates compound transport between compartments and introduces physiologically relevant permeability constraints. A drug introduced into the circulating medium distributes across organ modules according to relative flow fractions and tissue-specific uptake characteristics, thereby generating system-level PK behavior emerging from inter-organ interaction [119].

A central objective of this approach is quantitative prediction of human drug PK profiles under physiologically scaled conditions [114]. Rather than prescribing concentration–time inputs, the platform measures circulating drug levels over time and compares the resulting trajectories with clinical plasma concentration data. Clearance, half-life, and tissue distribution parameters derived from the chip are evaluated against known human PK values to assess predictive fidelity [118].

A distinguishing feature of vascularized systems lies in their integration with PBPK modeling frameworks [115]. Organ volumes, flow rates, and metabolic capacities within the chip are scaled according to human physiological ratios, allowing experimental data to be directly mapped onto PBPK model structures [117]. In this configuration, the organ-chip network functions as a reduced-scale physical analog of a human PBPK system, enabling empirical validation of model-derived predictions [118].

For selected compounds, these platforms suggest that human-relevant PK parameters can be approximated with reduced reliance on animal-derived scaling factors [114]. By using primary human cells and physiologically proportioned inter-organ flow distributions, the system reduces uncertainties associated with interspecies extrapolation. Drug-specific clearance and distribution behaviors emerge from coordinated hepatic metabolism, renal elimination, and tissue partitioning processes within the vascularized network [59].

The predictive performance of the platform is evaluated by comparing chip-derived PK curves to observed human clinical data. For selected compounds, concentration–time profiles generated within the vascularized multi-organ chip align within acceptable fold-error ranges relative to reported human plasma kinetics. Such comparisons provide experimental evidence that integrated MPSs can approximate systemic human PK with quantitative consistency [118].

From a translational standpoint, vascularized multi-organ platforms bridge the gap between isolated organ modeling and full systemic prediction [111]. By reconstructing circulation-driven drug disposition under physiologically scaled conditions, these systems move beyond emulation of predefined PK curves and toward endogenous generation of human-like PK responses. This capability positions vascularized MPSs as experimental counterparts to PBPK modeling, supporting iterative refinement between computational prediction and empirical measurement [115].

Technically, achieving predictive accuracy requires stable long-term recirculation, precise scaling of organ-to-organ flow ratios, and minimization of compound adsorption within microfluidic materials [119]. Endothelial barrier integrity and interfacial transport dynamics must also be carefully characterized, as vascular permeability influences effective tissue exposure. By integrating vascular transport, multi-organ interaction, and quantitative scaling, these platforms advance experimental prediction of human PK in vitro [118].

## 6. Integration with PBPK Modeling for Quantitative In Vitro–In Vivo Translation

While MPSs have progressively advanced from dynamic exposure control to multi-organ PK reconstruction, their translational value ultimately depends on integration with quantitative modeling frameworks [120]. The transition from experimentally measured in vitro parameters to predictive human PK requires a structured interface between organ-chip data and PBPK models [121]. Microphysiological platforms do not replace modeling but instead function as empirical parameter generators within a model-informed translational workflow [122].

A central distinction must be drawn between IVIVE and quantitative in vitro–in vivo translation (IVIVT) [123]. IVIVE traditionally refers to the scaling of intrinsic clearance or permeability parameters obtained from simplified systems—such as hepatocytes or microsomes—to whole-organ or whole-body estimates using predefined physiological scaling factors [124]. In contrast, IVIVT extends beyond parameter scaling and seeks to align full concentration–time relationships between in vitro systems and in vivo outcomes. Rather than extrapolating isolated kinetic constants, IVIVT evaluates whether temporally structured exposure–response relationships observed in MPSs can predict systemic PK behavior under clinically relevant conditions [121].

Within this framework, organ-chip platforms serve distinct roles depending on their level of physiological integration (Table 1) [122]. Dynamic intestinal models provide time-resolved absorption rate constants that can be incorporated as ka inputs within PBPK structures [103]. Human Duodenum Chips refine this contribution by integrating transporter expression and metabolic competence, enabling mechanistic estimation of intestinal first-pass extraction [108]. Genome-edited intestine–liver systems further reconstruct sequential absorption and hepatic metabolism, generating composite first-pass parameters under programmable concentration–time profiles [110].

At the systemic level, multi-tissue microphysiological platforms contribute experimentally derived intrinsic clearance (CL_int_), distribution kinetics, and inter-organ interaction parameters [112,125]. When appropriately normalized for cell number, organ scaling, and flow fraction, these measurements can populate PBPK models with empirically determined values rather than literature-derived approximations. Vascularized multi-organ systems extend this integration by reproducing circulation-driven drug disposition within physiologically proportioned networks [118]. In such configurations, the organ-chip platform operates as a reduced-scale physical analog of a PBPK system, enabling direct comparison between chip-generated and clinically observed concentration–time trajectories [121].

PK-informed MPSs should be considered complementary to established pharmacokinetic and modeling approaches rather than replacements for them. Conventional in vivo PK studies remain essential for confirming systemic exposure but are limited by species differences and reduced control over individual mechanisms. PBPK and physiologically based biopharmaceutics modeling (PBBM) provide mechanistic simulation frameworks for systemic disposition and formulation-dependent absorption, whereas IVIVE and IVIVC support parameter scaling or formulation–exposure relationships. In contrast, PK-informed MPSs provide experimentally controlled, human-cell-based dynamic exposure, transport, metabolism, and response data that can inform these models, although their predictive value depends on platform validation, physiological scaling, and correction of material-related artifacts.

For example, a recent pharmacokinetic exposure/microfluidic platform study directly compared in vivo, in silico, and platform-derived outputs for paclitaxel-loaded polymeric micelles, providing a representative case in which predicted versus observed exposure profiles and formulation-dependent pharmacodynamic responses could be evaluated quantitatively. Such studies demonstrate how PK-informed MPSs can support quantitative model qualification beyond qualitative in vitro readouts [126].

Quantitative IVIVT, therefore, emerges not as a unidirectional extrapolation but as an iterative alignment process between experiment and model [120,127]. Chip-derived PK outputs are mapped onto PBPK simulations, discrepancies are analyzed to refine scaling assumptions, and revised model predictions can subsequently be re-implemented experimentally within programmable microfluidic systems [121]. This bidirectional loop reduces uncertainty associated with interspecies extrapolation and enhances parameter identifiability by constraining model space with empirical human-cell-based data.

A critical component of successful integration lies in physiologically consistent scaling. Organ-to-organ flow ratios, relative tissue volumes, and metabolic capacity must be proportionally represented within the microfluidic architecture [128,129]. Deviations from physiological scaling can distort emergent PK behavior and compromise predictive fidelity [129]. Moreover, compound adsorption to device materials, nonspecific binding, and incomplete recirculation stability introduce systematic biases that must be quantitatively characterized before PBPK mapping [123].

Another consideration involves defining acceptable predictive thresholds. Rather than requiring exact replication of clinical PK curves, practical IVIVT frameworks often evaluate performance within defined fold-error margins [124]. Such criteria acknowledge biological variability and model uncertainty while preserving quantitative rigor. MPSs offer the advantage of isolating kinetic variables from confounding species-specific physiology, thereby improving the interpretability of scaling discrepancies [120].

By integrating dynamic exposure engineering, organ-level interaction, and PBPK modeling, MPSs evolve from descriptive in vitro assays into quantitative translational tools [122]. The convergence of programmable PK control and physiologically structured multi-organ interaction enables reconstruction of systemic drug disposition under human-relevant conditions [115]. In this model-informed paradigm, organ-chip platforms provide mechanistically grounded input parameters, PBPK frameworks supply structural organization, and IVIVT serves as the evaluative bridge linking microscale experimentation to clinical PK prediction [121].

Together, these developments position PK-informed MPSs as experimental counterparts to systems pharmacology modeling [120]. Rather than operating in parallel, organ-chip experimentation and PBPK simulation become mutually constraining components of a unified translational strategy [124]. This integration provides a pathway by which dynamic in vitro PK measurements may be systematically extended toward human PK prediction.

From a regulatory perspective, PK-informed MPS data are likely to be most useful as supportive, fit-for-purpose evidence for PBPK model parameterization, mechanistic interpretation, and exposure-based hypothesis testing rather than as stand-alone replacements for conventional PK studies. Acceptance will depend on analytical validation, reproducibility, and comparison of MPS-derived predictions with observed preclinical or clinical PK data [126].

## 7. Current Limitations and Future Directions

Despite substantial advances in dynamic exposure control, multi-organ integration, and quantitative PBPK coupling, several technical and conceptual challenges continue to limit the predictive reliability of MPSs for systemic PK reconstruction (Table 2) [130]. Addressing these limitations is essential for transforming organ-chip platforms from experimental tools into standardized translational infrastructures [131].

One fundamental challenge arises from fluidic linking artifacts inherent to interconnected microfluidic systems [129]. In multi-organ configurations, intercompartmental flow rates and volume ratios must approximate physiological scaling relationships [132]. However, deviations in channel resistance, recirculation efficiency, or dead volume can distort effective residence time and concentration gradients. Such distortions may alter emergent PK behavior independently of intrinsic metabolic or transport properties [128]. Without rigorous validation of fluidic stability and scaling proportionality, systemic clearance and distribution parameters derived from these systems may reflect architectural bias rather than biological kinetics [133].

Protein binding represents an additional source of uncertainty in translating chip-derived PK parameters [134]. In vivo, only the unbound fraction of drug contributes to distribution, metabolism, and elimination [135]. MPSs frequently operate under serum-reduced or protein-controlled conditions, potentially altering free fraction dynamics relative to human plasma. Inaccurate accounting of protein binding may lead to overestimation or underestimation of intrinsic clearance when mapping experimental data onto PBPK frameworks. Quantitative determination of free fraction within chip circulation compartments becomes essential for reliable in vitro–in vivo alignment [135].

Material adsorption, particularly in devices fabricated from PDMS, further complicates quantitative interpretation [136]. Lipophilic compounds may partition into polymeric materials, resulting in time-dependent depletion of circulating drug concentration that is unrelated to biological clearance [30,31]. Although alternative materials and surface treatments have been introduced, adsorption effects remain compound-specific and must be empirically characterized for each system. Failure to correct for adsorption artifacts can distort estimated elimination kinetics and compromise IVIVT accuracy [136].

Another limitation concerns the incomplete representation of immune and inflammatory components within current microphysiological architectures [137]. Many drug disposition processes—especially for biologics or oncology therapeutics—are influenced by immune-mediated clearance, cytokine signaling, or inflammation-induced enzyme modulation [138]. The absence of immune cell populations may, therefore, limit predictive fidelity in scenarios where PK behavior is coupled to immunological dynamics. Integration of immune-relevant modules remains technically challenging but increasingly necessary for comprehensive translational modeling [139,140].

Tumor heterogeneity presents an additional layer of complexity when applying PK-informed platforms to oncology [87]. Even within vascularized or spatially resolved systems, microenvironmental gradients in oxygenation, stromal composition, and cellular phenotype may not fully recapitulate in vivo tumor diversity. As a consequence, PK–PD coupling observed in chip-based tumor models may represent a subset of clinically relevant exposure–response variability. Standardized metrics for spatial scaling and microenvironmental representation are required to improve reproducibility across platforms [131].

Beyond biological and material considerations, inter-laboratory variability and the absence of harmonized scaling criteria remain critical barriers to widespread adoption [132]. Differences in cell sourcing, passage number, flow calibration, and analytical quantification methods can introduce variability that complicates cross-study comparison [133]. Establishing standardized reporting guidelines for flow ratios, scaling assumptions, adsorption correction methods, and validation metrics would enhance reproducibility and facilitate regulatory evaluation [130].

Importantly, many of these limitations converge on a common issue: parameter uncertainty within quantitative translation [131]. Whether arising from fluidic distortion, protein binding misestimation, material interaction, or incomplete biological representation, each source of variability ultimately affects the mapping between chip-derived parameters and PBPK predictions [138]. Addressing these uncertainties requires systematic benchmarking, transparent scaling methodology, and integration of experimental variability into model confidence intervals [133].

Standardization efforts should, therefore, prioritize three interconnected domains: architectural scaling consistency, biochemical exposure accuracy, and analytical validation rigor [132]. Only through harmonized methodological frameworks can MPSs fulfill their potential as quantitative contributors to predictive human PK [120].

## 8. Discussion

From a practical drug development perspective, PK-informed MPSs may support decision-making by linking dynamic exposure profiles to experimentally observed efficacy, toxicity, absorption, metabolism, and tissue distribution outcomes. These data may help prioritize compounds, compare dosing schedules or formulations, assess drug–drug interaction (DDI) or food-effect risks, and refine PBPK/PBBM simulations. At later stages, MPS-derived evidence may provide supportive mechanistic information for first-in-human dose rationale or model-based regulatory justification, while remaining complementary to conventional PK and clinical studies.

These platforms may also support formulation-related decision-making across development stages, including selection of formulations for preclinical toxicity studies, first-in-human evaluation, later clinical development, and final product optimization. In addition, PK-informed intestinal or intestine–liver MPSs, particularly when combined with PBPK/PBBM modeling, may provide mechanistic information for salt-form/formulation screening, DDI and food-effect assessment, and bioequivalence-related formulation risk evaluation. However, these applications should currently be viewed as complementary tools and require further validation before routine regulatory use.

To realize these applications more broadly, future progress will likely depend on tighter integration between experimental platforms, computational modeling, and data-driven inference [141,142]. Multi-organ MPSs generate high-dimensional datasets, including time-resolved concentration profiles, metabolic fluxes, and tissue-specific response metrics [143]. Artificial intelligence (AI)-assisted approaches may help identify nonlinear relationships among these variables, estimate intrinsic clearance and scaling parameters, and support calibration of PBPK models under conditions of experimental uncertainty [142,144]. Such approaches could facilitate iterative comparison between experimentally observed and model-predicted PK behavior while reducing reliance on manual parameter adjustment.

Digital twin architectures represent a related opportunity for extending MPS-derived data toward individualized PK prediction [145,146]. Patient-specific physiological characteristics, including organ volumes, blood-flow distributions, enzyme expression levels, and genetic variability, can be incorporated into computational models constrained by experimentally measured MPS data. In this framework, MPSs provide mechanistic experimental inputs, whereas digital twins extrapolate these observations toward patient-level exposure and response. Iterative updating between experimental and computational components may ultimately enable evaluation of individualized dosing strategies under defined physiological conditions.

Ultimately, the convergence of programmable microfluidic systems, quantitative PBPK modeling, and AI-assisted inference defines a pathway toward predictive systems pharmacology [143]. Rather than operating as isolated experimental tools, MPSs are poised to become integral components of an integrated digital–experimental ecosystem [144]. In this paradigm, dynamic in vitro PK measurements inform computational models, which in turn guide experimental redesign in an iterative cycle of refinement.

By embedding microphysiological platforms within such a digitally augmented framework, the field moves closer to predictive human PK grounded in mechanistic experimentation rather than empirical extrapolation [120]. This evolution from static potency assessment to AI-enabled, model-informed translation represents the logical culmination of PK-informed microphysiological system development [141,147].

## 9. Conclusions

MPSs have evolved from static cell-based assays toward dynamic, programmable platforms capable of reconstructing PK behavior under physiologically structured conditions. By engineering concentration–time profiles in vitro, integrating organ-level interactions, and coupling experimental outputs with quantitative PBPK modeling, these systems extend beyond descriptive pharmacology into the domain of predictive translational science.

A central shift highlighted in this review is the transition from static potency metrics to time-resolved PK–PD coupling. Dynamic exposure control, multi-compartment integration, and vascularized circulation architectures collectively enable reconstruction of systemic drug disposition that more closely approximates human physiology. When embedded within a model-informed framework, organ-chip platforms function not merely as exposure emulators but as empirical contributors to parameterized human PK prediction.

Despite these advances, several limitations remain that constrain the predictive reliability of current MPSs. Limitations in scaling fidelity, material compatibility, protein binding, and biological completeness can distort effective exposure levels within microfluidic circulation. In addition, incomplete representation of immune and inflammatory processes may limit the ability of existing platforms to capture PK behaviors associated with complex therapeutics. Addressing these challenges will require improved material engineering, standardized scaling frameworks, and systematic benchmarking against clinical PK datasets.

Ultimately, PK-informed MPSs represent a conceptual integration of experimental biology and systems pharmacology. Rather than relying solely on interspecies extrapolation or static in vitro endpoints, future translational workflows may increasingly depend on iterative alignment between microphysiological measurement and human-centered computational modeling. Such integration defines the trajectory by which dynamic in vitro PK data can be systematically extended toward predictive human drug disposition.

## Figures and Tables

**Table 1:** Hierarchical roles of microphysiological systems in PBPK integration and quantitative IVIVT. Representative PK-informed microphysiological platforms are compared according to their typical experimental readouts, translated PK parameters, PBPK/IVIVT roles, strengths, and limitations.

Framework/Platform	Typical Experimental Readout	Translated Parameter or PK Quantity	PBPK/IVIVT Role	Strength	Limitation
**IVIVE**	Simplified in vitro kinetic constants from hepatocytes, microsomes, or permeability assays	CL_int_, Papp, permeability-related constants	Parameter scaling to organ or whole-body estimates using predefined physiological factors	Simple and widely established scaling framework	Limited temporal structure and weak representation of dynamic exposure biology
**Quantitative IVIVT**	Time-resolved concentration–time and exposure–response relationships from dynamic MPSs	Exposure-linked absorption, clearance, first-pass, or system-level PK relationships	Iterative alignment of chip-derived and in vivo/clinical PK trajectories	Captures dynamic behavior and supports bidirectional experiment–model refinement	Requires rigorous scaling, analytical validation, and clear acceptance criteria
**Dynamic intestinal model**	Time-resolved transepithelial flux, effective absorption rate, permeability under flow	Intestinal absorption kinetics, ka-related inputs, dynamic permeability terms	Upstream absorption parameterization under non-equilibrium transport conditions	Captures luminal concentration decay and flow-dependent transport behavior	Limited epithelial complexity and incomplete representation of intestinal metabolism
**Human Duodenum Chip**	Transporter expression/activity, CYP3A4 function, absorbed and metabolized fractions under perfusion	Intestinal absorption plus mechanistic first-pass extraction	Mechanistic intestinal module for PBPK absorption and gut-wall metabolism	Human-relevant epithelial phenotype and metabolic competence	Donor variability, technical complexity, and long-term reproducibility challenges
**Intestine–liver system**	Sequential transepithelial transport, hepatic parent–metabolite conversion, first-pass extraction under perfusion	Composite first-pass parameters integrating absorption and hepatic metabolism	Sequential gut–liver first-pass parameterization	Captures coordinated portal transfer and metabolism	Requires stable recirculation and physiologically consistent inter-organ flow scaling
**Multi-tissue MPS**	Time-resolved circulating concentrations, organ-specific clearance contributions, inter-organ interaction parameters	Systemic clearance, distribution kinetics, and intercompartmental PK behavior	Systemic disposition parameterization for PBPK-informed IVIVT	Reconstructs organ-interaction-driven PK rather than isolated organ outputs	Sensitive to organ scaling, dead volume, and compound adsorption
**Vascularized multi-organ MPS**	Circulation-driven concentration–time trajectories, tissue distribution behavior, vascular transport under scaled flow	Human-like systemic PK reconstruction, including clearance, half-life, and distribution behavior	Reduced-scale physical analog for PBPK validation against clinical PK trajectories	Highest physiological integration and strongest bridge to human PK prediction	High technical burden, endothelial stability requirements, and scaling sensitivity

**Table 2:** Major limitations affecting predictive PK translation in microphysiological systems. Key technical and biological sources of uncertainty are summarized together with their translational consequences and future directions for improving PBPK/IVIVT reliability.

Limitation Domain	Primary Source of Error	Translational Consequence	Future Direction
**Fluidic linking and scaling**	Non-physiological flow ratios, channel resistance, dead volume, unstable recirculation	Clearance and distribution behavior may reflect device architecture rather than intrinsic biology, weakening PBPK/IVIVT reliability	Standardized scaling rules, recirculation validation, dead-volume correction
**Material adsorption**	Compound loss to PDMS or other device materials	Apparent elimination may be overestimated and incorrectly interpreted as metabolism or tissue uptake	Compound-specific adsorption testing, alternative materials, correction models
**Protein binding**	Non-physiological protein conditions alter unbound fraction	Free-drug exposure, intrinsic clearance, and tissue distribution may be misestimated during PBPK mapping	Quantification of unbound fraction in chip circulation compartments
**Incomplete biological representation**	Limited immune and inflammatory components	Predictive fidelity decreases for biologics, oncology drugs, and inflammation-sensitive PK processes	Integration of immune-relevant modules and cytokine-responsive systems
**Tumor microenvironment heterogeneity**	Incomplete representation of oxygen, stromal, and phenotypic gradients	Spatial exposure–response variability may be underestimated, limiting oncology translation	Standardized spatial metrics and more physiologically representative architectures
**Inter-laboratory variability**	Differences in cell source, passage, flow calibration, and analytics	Cross-study reproducibility declines, making benchmarking and regulatory interpretation difficult	Harmonized reporting guidelines and benchmarking studies
**Parameter uncertainty in translation**	Technical and biological uncertainties propagate into model inputs	Confidence in PBPK prediction and quantitative IVIVT decreases, even when local chip data appear robust	Confidence intervals, sensitivity analysis, and uncertainty-aware model integration
**Need for standardization**	Lack of common criteria for scaling, exposure accuracy, and validation	Broader adoption, cross-platform comparison, and regulatory acceptance remain limited	Consensus frameworks for architectural scaling, biochemical accuracy, and analytical validation

## Data Availability

Data availability is not applicable to this article because no new data were generated or analyzed.

## Abbreviations

The following abbreviations are used in this manuscript:

AI	artificial intelligence
AUC	area under the concentration–time curve
CC-3	cleaved caspase-3
CL	clearance
CLint	intrinsic clearance
ClpP	caseinolytic protease P
C_max_	maximum concentration
CYP	cytochrome P450
CYP3A4	cytochrome P450 3A4
E_max_	maximum effect
IC_50_	half-maximal inhibitory concentration
IVIVE	in vitro–in vivo extrapolation
IVIVT	in vitro–in vivo translation
K_a_	absorption rate constant
LC–MS/MS	liquid chromatography–tandem mass spectrometry
MALDI	matrix-assisted laser desorption/ionization
MIC	minimum inhibitory concentration
MPS	microphysiological systems
MSI	mass spectrometry imaging
Papp	apparent permeability coefficient
PBPK	physiologically based pharmacokinetic
PD	pharmacodynamics
PDMS	polydimethylsiloxane
PK	pharmacokinetics
PXR	pregnane X receptor
RIF	rifampicin
SILAC	stable isotope labeling by amino acids in cell culture
t_1_/_2_	elimination half-life
T_max_	time to maximum concentration
UGT1A1	uridine 5′-diphospho-glucuronosyltransferase 1A1
VD3	vitamin D3
Vd	volume of distribution
VDR	vitamin D receptor
γH2AX	phosphorylated histone H2AX
DDI	drug–drug interaction
PBBM	physiologically based biopharmaceutics modeling

## References

[1] Sánchez-Díez M., Romero-Jiménez P., Alegría-Aravena N., Gavira-O’Neill C.E., Vicente-García E., Quiroz-Troncoso J., González-Martos R., Ramírez-Castillejo C., Pastor J.M. (2025). Assessment of cell viability in drug therapy: IC50 and other new time-independent indices for evaluating chemotherapy efficacy. Pharmaceutics.

[2] Hafner M., Niepel M., Sorger P.K. (2017). Alternative drug sensitivity metrics improve preclinical cancer pharmacogenomics. Nat. Biotechnol..

[3] Kim S., Hwang S. (2021). Preclinical drug response metric based on cellular response phenotype provides better pharmacogenomic variables with phenotype relevance. Pharmaceuticals.

[4] Holford N. (2018). Pharmacodynamic principles and the time course of delayed and cumulative drug effects. Transl. Clin. Pharmacol..

[5] Karlsson M.O., Molnar V., Bergh J., Freijs A., Larsson R. (1998). A general model for time-dissociated pharmacokinetic-pharmacodynamic relationships exemplified by paclitaxel myelosuppression. Clin. Pharmacol. Ther..

[6] Matsushima Y., Kanzawa F., Hoshi A., Shimizu E., Nomori H., Sasaki Y., Saijo N. (1985). Time-schedule dependency of the inhibiting activity of various anticancer drugs in the clonogenic assay. Cancer Chemother. Pharmacol..

[7] Evans D.M., Fang J., Silvers T., Delosh R., Laudeman J., Ogle C., Reinhart R., Selby M., Bowles L., Connelly J. (2019). Exposure time versus cytotoxicity for anticancer agents. Cancer Chemother. Pharmacol..

[8] Chen S.-h., Forrester W., Lahav G. (2016). Schedule-dependent interaction between anticancer treatments. Science.

[9] Howard G.R., Jost T.A., Yankeelov T.E., Brock A. (2022). Quantification of long-term doxorubicin response dynamics in breast cancer cell lines to direct treatment schedules. PLoS Comput. Biol..

[10] Lohasz C., Loretan J., Sterker D., Görlach E., Renggli K., Argast P., Frey O., Wiesmann M., Wartmann M., Rausch M. (2021). A microphysiological cell-culturing system for pharmacokinetic drug exposure and high-resolution imaging of arrays of 3D microtissues. Front. Pharmacol..

[11] Komen J., Westerbeek E.Y., Kolkman R.W., Roesthuis J., Lievens C., Van Den Berg A., Van Der Meer A.D. (2020). Controlled pharmacokinetic anti-cancer drug concentration profiles lead to growth inhibition of colorectal cancer cells in a microfluidic device. Lab A Chip.

[12] Guerrero Y.A., Desai D., Sullivan C., Kindt E., Spilker M.E., Maurer T.S., Solomon D.E., Bartlett D.W. (2020). A microfluidic perfusion platform for in vitro analysis of drug pharmacokinetic-pharmacodynamic (PK-PD) relationships. AAPS J..

[13] McAleer C.W., Pointon A., Long C.J., Brighton R.L., Wilkin B.D., Bridges L.R., Narasimhan Sriram N., Fabre K., McDougall R., Muse V.P. (2019). On the potential of in vitro organ-chip models to define temporal pharmacokinetic-pharmacodynamic relationships. Sci. Rep..

[14] Danhof M., de Jongh J., De Lange E.C., Della Pasqua O., Ploeger B.A., Voskuyl R.A. (2007). Mechanism-based pharmacokinetic-pharmacodynamic modeling: Biophase distribution, receptor theory, and dynamical systems analysis. Annu. Rev. Pharmacol. Toxicol..

[15] Sung J.H., Esch M.B., Shuler M.L. (2010). Integration of in silico and in vitro platforms for pharmacokinetic–pharmacodynamic modeling. Expert Opin. Drug Metab. Toxicol..

[16] Abaci H.E., Shuler M.L. (2015). Human-on-a-chip design strategies and principles for physiologically based pharmacokinetics/pharmacodynamics modeling. Integr. Biol..

[17] Prantil-Baun R., Novak R., Das D., Somayaji M.R., Przekwas A., Ingber D.E. (2018). Physiologically based pharmacokinetic and pharmacodynamic analysis enabled by microfluidically linked organs-on-chips. Annu. Rev. Pharmacol. Toxicol..

[18] Edington C.D., Chen W.L.K., Geishecker E., Kassis T., Soenksen L.R., Bhushan B.M., Freake D., Kirschner J., Maass C., Tsamandouras N. (2018). Interconnected microphysiological systems for quantitative biology and pharmacology studies. Sci. Rep..

[19] Tsamandouras N., Chen W.L.K., Edington C.D., Stokes C.L., Griffith L.G., Cirit M. (2017). Integrated gut and liver microphysiological systems for quantitative in vitro pharmacokinetic studies. AAPS J..

[20] Erickson P., Jetley G., Amin P., Mejevdiwala A., Patel A., Cheng K., Parekkadan B. (2023). A cell culture system to model pharmacokinetics using adjustable-volume perfused mixing chambers. Toxicol. Vitr..

[21] Kutscher H.L., Morse G.D., Prasad P.N., Reynolds J.L. (2019). In vitro Pharmacokinetic Cell Culture System that Simulates Physiologic Drug and Nanoparticle Exposure to Macrophages: Kutscher, Morse, Prasad and Reynolds. Pharm. Res..

[22] Erickson P., Doshi A., Jetley G., Amin P., Mejevdiwala A., Patel A., Bento R., Parekkadan B. (2022). Multi-stream perfusion bioreactor integrated with outlet fractionation for dynamic cell culture. J. Vis. Exp..

[23] Lohasz C., Frey O., Bonanini F., Renggli K., Hierlemann A. (2019). Tubing-free microfluidic microtissue culture system featuring gradual, in vivo-like substance exposure profiles. Front. Bioeng. Biotechnol..

[24] Gimmel S., Maurer H.R. (1994). Growth kinetics of L1210 leukemic cells exposed to different concentration courses of methotrexate in vitro. Cancer Chemother. Pharmacol..

[25] He H., Cao Y. (2017). Chemotherapeutic dosing implicated by pharmacodynamic modeling of in vitro cytotoxic data: A case study of paclitaxel. J. Pharmacokinet. Pharmacodyn..

[26] Chen X., Li T., Zeng H., Hu Z., Fu B. (2016). Numerical and experimental investigation on micromixers with serpentine microchannels. Int. J. Heat Mass Transf..

[27] Arockiam S., Cheng Y.H., Armenante P.M., Basuray S. (2021). Experimental determination and computational prediction of the mixing efficiency of a simple, continuous, serpentine-channel microdevice. Chem. Eng. Res. Des..

[28] Minichmayr I.K., Kappetein S., Brill M.J., Friberg L.E. (2022). Model-informed translation of in vitro effects of short-, prolonged-and continuous-infusion meropenem against Pseudomonas aeruginosa to clinical settings. Antibiotics.

[29] Vogus D.R., Pusuluri A., Chen R., Mitragotri S. (2018). Schedule dependent synergy of gemcitabine and doxorubicin: Improvement of in vitro efficacy and lack of in vitro-in vivo correlation. Bioeng. Transl. Med..

[30] Grant J., Özkan A., Oh C., Mahajan G., Prantil-Baun R., Ingber D.E. (2021). Simulating drug concentrations in PDMS microfluidic organ chips. Lab A Chip.

[31] Carius P., Weinelt F.A., Cantow C., Holstein M., Teitelbaum A.M., Cui Y. (2024). Addressing the ADME challenges of compound loss in a PDMS-based gut-on-chip microphysiological system. Pharmaceutics.

[32] Van Meer B., de Vries H., Firth K., van Weerd J., Tertoolen L., Karperien H., Jonkheijm P., Denning C., IJzerman A., Mummery C. (2017). Small molecule absorption by PDMS in the context of drug response bioassays. Biochem. Biophys. Res. Commun..

[33] Grindulis K., Matusevica N.G., Kozlova V., Rimsa R., Klavins K., Mozolevskis G. (2025). Sorption and release of small molecules in PDMS and COC for Organs on chip. Sci. Rep..

[34] Auner A.W., Tasneem K.M., Markov D.A., McCawley L.J., Hutson M.S. (2019). Chemical-PDMS binding kinetics and implications for bioavailability in microfluidic devices. Lab A Chip.

[35] Kimura H., Nakamura H., Goto T., Uchida W., Uozumi T., Nishizawa D., Shinha K., Sakagami J., Doi K. (2024). Standalone cell culture microfluidic device-based microphysiological system for automated cell observation and application in nephrotoxicity tests. Lab A Chip.

[36] Orecchio F.M., Tommaso V., Santaniello T., Castiglioni S., Pezzotta F., Monti A., Butera F., Maier J.A.M., Milani P. (2022). A novel fluidic platform for semi-automated cell culture into multiwell-like bioreactors. Micromachines.

[37] Oduah E.I., Linhardt R.J., Sharfstein S.T. (2016). Heparin: Past, present, and future. Pharmaceuticals.

[38] Soenksen L.R., Kassis T., Noh M., Griffith L.G., Trumper D.L. (2018). Closed-loop feedback control for microfluidic systems through automated capacitive fluid height sensing. Lab A Chip.

[39] Murai J., Abdelmoez M.N., Kondo K., Takamuro K., Nozaki K., Schiller T., Scheibel T.R., Numata K., Yajima H., Kimura K.T. (2025). An open source for multiplexed, stable and transient flows to advance life sciences using microfluidic control automation. Lab A Chip.

[40] Schuster B., Junkin M., Kashaf S.S., Romero-Calvo I., Kirby K., Matthews J., Weber C.R., Rzhetsky A., White K.P., Tay S. (2020). Automated microfluidic platform for dynamic and combinatorial drug screening of tumor organoids. Nat. Commun..

[41] Jeong Y., Mercado-Vasquez G., Padhi A., Deng Y., Prakash M., Matthews J.M., Ozcan S., Toprak E., Tay S. (2025). Massively multiplexed microfluidics maps combinatorial and sequential antibiotic responses in 3D. bioRxiv.

[42] Chou W.-C., Lin Z. (2023). Machine learning and artificial intelligence in physiologically based pharmacokinetic modeling. Toxicol. Sci..

[43] Singh D., Deosarkar S.P., Cadogan E., Flemington V., Bray A., Zhang J., Reiserer R.S., Schaffer D.K., Gerken G.B., Britt C.M. (2022). A microfluidic system that replicates pharmacokinetic (PK) profiles in vitro improves prediction of in vivo efficacy in preclinical models. PLoS Biol..

[44] Garrison J., Li Z., Palanisamy B., Wang L., Seker E. (2018). An electrically-controlled programmable microfluidic concentration waveform generator. J. Biol. Eng..

[45] Guo Y., Deng P., Chen W., Li Z. (2020). Modeling pharmacokinetic profiles for assessment of anti-cancer drug on a microfluidic system. Micromachines.

[46] Elgack M.E., Abdelgawad M. (2025). Characterization of the dynamic flow response in microfluidic devices. Small Methods.

[47] Ramadan Q., Hazaymeh R., Zourob M. (2025). A Versatile and Modular Microfluidic System for Dynamic Cell Culture and Cellular Interactions. Micromachines.

[48] Hines D.E., Bell S., Chang X., Mansouri K., Allen D., Kleinstreuer N. (2022). Application of an accessible interface for pharmacokinetic modeling and in vitro to in vivo extrapolation. Front. Pharmacol..

[49] Gonzalez-Suarez A.M., Peña-del Castillo J.G., Hernández-Cruz A., Garcia-Cordero J.L. (2018). Dynamic generation of concentration-and temporal-dependent chemical signals in an integrated microfluidic device for single-cell analysis. Anal. Chem..

[50] Carvalho V., Gonçalves I.M., Rodrigues N., Sousa P., Pinto V., Minas G., Kaji H., Shin S.R., Rodrigues R.O., Teixeira S.F. (2024). Numerical evaluation and experimental validation of fluid flow behavior within an organ-on-a-chip model. Comput. Methods Programs Biomed..

[51] Zhao Y., Zhang K., Guo F., Yang M. (2021). Dynamic Modeling and Flow Distribution of Complex Micron Scale Pipe Network. Micromachines.

[52] Park S., Song S.-S., Jung W., Yun H.-y., Lee S., Kang S.-W., Chae J.-W. (2025). Mechanistic in vitro–in vivo extrapolation (IVIVE) approach using a biomimetic in vitro system for the prediction of hepatic clearance. Comput. Struct. Biotechnol. J..

[53] Fowler S., Chen W.L.K., Duignan D.B., Gupta A., Hariparsad N., Kenny J.R., Lai W.G., Liras J., Phillips J.A., Gan J. (2020). Microphysiological systems for ADME-related applications: Current status and recommendations for system development and characterization. Lab A Chip.

[54] Sung J.H., Wang Y., Shuler M.L. (2019). Strategies for using mathematical modeling approaches to design and interpret multi-organ microphysiological systems (MPS). APL Bioeng..

[55] Yu J., Cilfone N., Large E., Sarkar U., Wishnok J., Tannenbaum S., Hughes D., Lauffenburger D., Griffith L., Stokes C. (2015). Quantitative systems pharmacology approaches applied to microphysiological systems (MPS): Data interpretation and multi-MPS integration. CPT Pharmacomet. Syst. Pharmacol..

[56] Vasconez Martinez M.G., Frauenlob M., Rothbauer M. (2024). An update on microfluidic multi-organ-on-a-chip systems for reproducing drug pharmacokinetics: The current state-of-the-art. Expert Opin. Drug Metab. Toxicol..

[57] Sung J.H., Kam C., Shuler M.L. (2010). A microfluidic device for a pharmacokinetic–pharmacodynamic (PK–PD) model on a chip. Lab A Chip.

[58] Prot J.M., Maciel L., Bricks T., Merlier F., Cotton J., Paullier P., Bois F.Y., Leclerc E. (2014). First pass intestinal and liver metabolism of paracetamol in a microfluidic platform coupled with a mathematical modeling as a means of evaluating ADME processes in humans. Biotechnol. Bioeng..

[59] Novak R., Ingram M., Marquez S., Das D., Delahanty A., Herland A., Maoz B.M., Jeanty S.S., Somayaji M.R., Burt M. (2020). Robotic fluidic coupling and interrogation of multiple vascularized organ chips. Nat. Biomed. Eng..

[60] Choi S., Yim D.-s., Han S., Lee J. Integrated Microphysiological System and PBPK Modeling for Prediction of Human Diclofenac Pharmacokinetics. Proceedings of the American Conference of Pharmacometrics.

[61] Bucher R.W., Graves L.M., Bartlett D.W. (2025). Dose optimization of ClpP agonists using an in vitro microfluidic perfusion platform and in silico pharmacokinetic-pharmacodynamic modeling. AAPS J..

[62] Checkley S., MacCallum L., Yates J., Jasper P., Luo H., Tolsma J., Bendtsen C. (2015). Bridging the gap between in vitro and in vivo: Dose and schedule predictions for the ATR inhibitor AZD6738. Sci. Rep..

[63] El-Kareh A.W., Secomb T.W. (2003). A mathematical model for cisplatin cellular pharmacodynamics. Neoplasia.

[64] Yates J.W., Mistry H.B. (2023). Skipping a pillar does not make for strong foundations: Pharmacokinetic-pharmacodynamic reasoning behind the shape of dose–response relationships in oncology. CPT Pharmacomet. Syst. Pharmacol..

[65] Gabrielsson J., Andersson R., Jirstrand M., Hjorth S. (2019). Dose-response-time data analysis: An underexploited trinity. Pharmacol. Rev..

[66] Chen E.P., Dutta S., Ho M.-H., DeMartino M.P. (2024). Model-based virtual PK/PD exploration and machine learning approach to define PK drivers in early drug discovery. J. Med. Chem..

[67] Zhong Y., Huang M., Cao X., Lin R.-Y., Zhang Z.-Y., Zhu Q.-Q., Lei Y., Liu A.-L. (2026). Micro pharmacokinetics-pharmacodynamics monitoring of anti-Parkinson’s disease drugs using a microphysiological BBB-brain organ-on-a-chip. Microsyst. Nanoeng..

[68] Mistretta M., Gangneux N., Manina G. (2022). Microfluidic dose–response platform to track the dynamics of drug response in single mycobacterial cells. Sci. Rep..

[69] Zuieva A., Can S., Boelke F., Reuter S., Schattscheider S., Töpfer E., Westphal A., Mrowka R., Wölfl S. (2022). Real-time monitoring of immediate drug response and adaptation upon repeated treatment in a microfluidic chip system. Arch. Toxicol..

[70] Wright D.F., Winter H.R., Duffull S.B. (2011). Understanding the time course of pharmacological effect: A PKPD approach. Br. J. Clin. Pharmacol..

[71] Thiemicke A., Neuert G. (2023). Rate thresholds in cell signaling have functional and phenotypic consequences in non-linear time-dependent environments. Front. Cell Dev. Biol..

[72] Ayyar V.S., Jusko W.J. (2020). Transitioning from basic toward systems pharmacodynamic models: Lessons from corticosteroids. Pharmacol. Rev..

[73] Sung K.E., Beebe D.J. (2014). Microfluidic 3D models of cancer. Adv. Drug Deliv. Rev..

[74] Daryaee F., Tonge P.J. (2019). Pharmacokinetic–pharmacodynamic models that incorporate drug–target binding kinetics. Curr. Opin. Chem. Biol..

[75] Shah S., D’Souza G.G. (2025). Modeling tumor microenvironment complexity in vitro: Spheroids as physiologically relevant tumor models and strategies for their analysis. Cells.

[76] Karolak A., Rejniak K.A. (2019). Micropharmacology: An in silico approach for assessing drug efficacy within a tumor tissue. Bull. Math. Biol..

[77] Beller N.C., Wang Y., Hummon A.B. (2023). Evaluating the pharmacokinetics and pharmacodynamics of chemotherapeutics within a spatial SILAC-labeled spheroid model system. Anal. Chem..

[78] Kobayashi Y., Hashizume H., Takiguchi S., Ji J., Kawano R., Koiwai K., Yamamoto H., Elbadawy M., Omatsu T., Abugomaa A. (2025). A microfluidics platform for simultaneous evaluation of sensitivity and side effects of anti-cancer drugs using a three-dimensional culture method. Sci. Rep..

[79] Spruill M.L., Maletic-Savatic M., Martin H., Li F., Liu X. (2022). Spatial analysis of drug absorption, distribution, metabolism, and toxicology using mass spectrometry imaging. Biochem. Pharmacol..

[80] Bareham B., Dibble M., Parsons M. (2024). Defining and modeling dynamic spatial heterogeneity within tumor microenvironments. Curr. Opin. Cell Biol..

[81] LaBonia G.J., Lockwood S.Y., Heller A.A., Spence D.M., Hummon A.B. (2016). Drug penetration and metabolism in 3D cell cultures treated in a 3D printed fluidic device: Assessment of irinotecan via MALDI imaging mass spectrometry. Proteomics.

[82] Trédan O., Galmarini C.M., Patel K., Tannock I.F. (2007). Drug resistance and the solid tumor microenvironment. J. Natl. Cancer Inst..

[83] Achilli T.-M., McCalla S., Meyer J., Tripathi A., Morgan J.R. (2014). Multilayer spheroids to quantify drug uptake and diffusion in 3D. Mol. Pharm..

[84] De Maar J.S., Sofias A.M., Siegel T.P., Vreeken R.J., Moonen C., Bos C., Deckers R. (2020). Spatial heterogeneity of nanomedicine investigated by multiscale imaging of the drug, the nanoparticle and the tumour environment. Theranostics.

[85] Lopez A., Holbrook J.H., Hummon A.B. (2025). MALDI imaging and spatial SILAC proteomics of three-dimensional multicellular spheroids dynamically dosed with doxorubicin-encapsulating liposomes. Anal. Chem..

[86] Tobias F., Hummon A.B. (2020). Considerations for MALDI-based quantitative mass spectrometry imaging studies. J. Proteome Res..

[87] Verloy R., Privat-Maldonado A., Van Audenaerde J., Rovers S., Zaryouh H., De Waele J., Quatannens D., Peeters D., Roeyen G., Deben C. (2025). Capturing the heterogeneity of the PDAC tumor microenvironment: Novel triple co-culture spheroids for drug screening and angiogenic evaluation. Cells.

[88] Rzagalinski I., Volmer D.A. (2017). Quantification of low molecular weight compounds by MALDI imaging mass spectrometry—A tutorial review. Biochim. ET Biophys. Acta (BBA)-Proteins Proteom..

[89] Liu X., Fang J., Huang S., Wu X., Xie X., Wang J., Liu F., Zhang M., Peng Z., Hu N. (2021). Tumor-on-a-chip: From bioinspired design to biomedical application. Microsyst. Nanoeng..

[90] Caballero D., Blackburn S.M., De Pablo M., Samitier J., Albertazzi L. (2017). Tumour-vessel-on-a-chip models for drug delivery. Lab A Chip.

[91] Petreus T., Cadogan E., Hughes G., Smith A., Pilla Reddy V., Lau A., O’Connor M.J., Critchlow S., Ashford M., Oplustil O’Connor L. (2021). Tumour-on-chip microfluidic platform for assessment of drug pharmacokinetics and treatment response. Commun. Biol..

[92] Avendano A., Cortes-Medina M., Song J.W. (2019). Application of 3-D microfluidic models for studying mass transport properties of the tumor interstitial matrix. Front. Bioeng. Biotechnol..

[93] Wang H.-F., Ran R., Liu Y., Hui Y., Zeng B., Chen D., Weitz D.A., Zhao C.-X. (2018). Tumor-vasculature-on-a-chip for investigating nanoparticle extravasation and tumor accumulation. ACS Nano.

[94] Cao X., Ashfaq R., Cheng F., Maharjan S., Li J., Ying G., Hassan S., Xiao H., Yue K., Zhang Y.S. (2019). A tumor-on-a-chip system with bioprinted blood and lymphatic vessel pair. Adv. Funct. Mater..

[95] Shirure V.S., Bi Y., Curtis M.B., Lezia A., Goedegebuure M.M., Goedegebuure S.P., Aft R., Fields R.C., George S.C. (2018). Tumor-on-a-chip platform to investigate progression and drug sensitivity in cell lines and patient-derived organoids. Lab A Chip.

[96] Trujillo-de Santiago G., Flores-Garza B.G., Tavares-Negrete J.A., Lara-Mayorga I.M., Gonzalez-Gamboa I., Zhang Y.S., Rojas-Martinez A., Ortiz-Lopez R., Alvarez M.M. (2019). The tumor-on-chip: Recent advances in the development of microfluidic systems to recapitulate the physiology of solid tumors. Materials.

[97] Youhanna S., Lauschke V.M. (2021). The past, present and future of intestinal in vitro cell systems for drug absorption studies. J. Pharm. Sci..

[98] Przybylla R., Mullins C.S., Krohn M., Oswald S., Linnebacher M. (2022). Establishment and characterization of novel human intestinal in vitro models for absorption and first-pass metabolism studies. Int. J. Mol. Sci..

[99] Asano S., Yoshitomo A., Hozuki S., Sato H., Kazuki Y., Hisaka A. (2021). A new intestinal model for analysis of drug absorption and interactions considering physiological translocation of contents. Drug Metab. Dispos..

[100] Imaoka T., Onuki-Nagasaki R., Kimura H., Tai K., Ishii M., Nozue A., Kaisaki I., Hoshi M., Watanabe K., Maeda K. (2024). Development of a novel gut microphysiological system that facilitates assessment of drug absorption kinetics in gut. Sci. Rep..

[101] Santbergen M.J., Van der Zande M., Gerssen A., Bouwmeester H., Nielen M.W. (2020). Dynamic in vitro intestinal barrier model coupled to chip-based liquid chromatography mass spectrometry for oral bioavailability studies. Anal. Bioanal. Chem..

[102] Palamà M.E.F., Aiello M., Borka G., Furci J., Parodi I., Firpo G., Scaglione S. (2025). A Dynamic Double-Flow Gut-On-Chip Model for Predictive Absorption Studies In Vitro. Adv. Mater. Technol..

[103] Keuper-Navis M., Walles M., Poller B., Myszczyszyn A., van der Made T.K., Donkers J., Amirabadi H.E., Wilmer M.J., Aan S., Spee B. (2023). The application of organ-on-chip models for the prediction of human pharmacokinetic profiles during drug development. Pharmacol. Res..

[104] Kimura H., Ikeda T., Nakayama H., Sakai Y., Fujii T. (2015). An on-chip small intestine–liver model for pharmacokinetic studies. J. Lab. Autom..

[105] Milani N., Parrott N., Franyuti D.O., Godoy P., Galetin A., Gertz M., Fowler S. (2022). Application of a gut–liver-on-a-chip device and mechanistic modelling to the quantitative in vitro pharmacokinetic study of mycophenolate mofetil. Lab A Chip.

[106] Kasendra M., Tovaglieri A., Sontheimer-Phelps A., Jalili-Firoozinezhad S., Bein A., Chalkiadaki A., Scholl W., Zhang C., Rickner H., Richmond C.A. (2018). Development of a primary human Small Intestine-on-a-Chip using biopsy-derived organoids. Sci. Rep..

[107] Kasendra M., Luc R., Yin J., Manatakis D.V., Kulkarni G., Lucchesi C., Sliz J., Apostolou A., Sunuwar L., Obrigewitch J. (2020). Duodenum Intestine-Chip for preclinical drug assessment in a human relevant model. Elife.

[108] Zagaja K.M.P., Kopec A.K., Harney J., Hardink M.A., Middleton J., Hens B. (2026). Establishing the Human Duodenum Chip as a Surrogate for Effective Human Permeability: In Vitro and In Silico Assessment. AAPS J..

[109] Marin T.M., de Carvalho Indolfo N., Rocco S.A., de Carvalho M., Dias M.M., Bento G.I.V., Bortot L.O., Schuck D.C., Lorencini M., Pagani E. (2020). An intestine/liver microphysiological system for drug pharmacokinetic and toxicological assessment. JoVE (J. Vis. Exp.).

[110] Negoro R., Deguchi S., Yamazaki D., Takayama K., Fujita T. (2025). Genome edited intestine liver on a chip system for integrated intestinal hepatic drug absorption and metabolism evaluation. Sci. Rep..

[111] Van Ness K.P., Cesar F., Yeung C.K., Himmelfarb J., Kelly E.J. (2022). Microphysiological systems in absorption, distribution, metabolism, and elimination sciences. Clin. Transl. Sci..

[112] Sherfey J., Rajan S.A.P., Nichol L., Parekh P., Smith J.T., Gregory L., Clark F., Ohri S., Kadar E.P., George B.T. (2025). Predicting Human Pharmacokinetic Parameters of Drugs using a Multi-Tissue Chip Platform Integrating Liver, Kidney, and Skeletal Muscle Microphysiological Systems. bioRxiv.

[113] Vernetti L., Gough A., Baetz N., Blutt S., Broughman J.R., Brown J.A., Foulke-Abel J., Hasan N., In J., Kelly E. (2017). Functional coupling of human microphysiology systems: Intestine, liver, kidney proximal tubule, blood-brain barrier and skeletal muscle. Sci. Rep..

[114] Isoherranen N., Madabushi R., Huang S.M. (2019). Emerging role of organ-on-a-chip technologies in quantitative clinical pharmacology evaluation. Clin. Transl. Sci..

[115] Yang Y., Chen Y., Wang L., Xu S., Fang G., Guo X., Chen Z., Gu Z. (2022). PBPK modeling on organs-on-chips: An overview of recent advancements. Front. Bioeng. Biotechnol..

[116] Imaoka T., Huang W., Shum S., Hailey D.W., Chang S.-Y., Chapron A., Yeung C.K., Himmelfarb J., Isoherranen N., Kelly E.J. (2021). Bridging the gap between in silico and in vivo by modeling opioid disposition in a kidney proximal tubule microphysiological system. Sci. Rep..

[117] Sung J.H., Srinivasan B., Esch M.B., McLamb W.T., Bernabini C., Shuler M.L., Hickman J.J. (2014). Using physiologically-based pharmacokinetic-guided “body-on-a-chip” systems to predict mammalian response to drug and chemical exposure. Exp. Biol. Med..

[118] Herland A., Maoz B.M., Das D., Somayaji M.R., Prantil-Baun R., Novak R., Cronce M., Huffstater T., Jeanty S.S., Ingram M. (2020). Quantitative prediction of human pharmacokinetic responses to drugs via fluidically coupled vascularized organ chips. Nat. Biomed. Eng..

[119] Ronaldson-Bouchard K., Teles D., Yeager K., Tavakol D.N., Zhao Y., Chramiec A., Tagore S., Summers M., Stylianos S., Tamargo M. (2022). A multi-organ chip with matured tissue niches linked by vascular flow. Nat. Biomed. Eng..

[120] Cirit M., Stokes C.L. (2018). Maximizing the impact of microphysiological systems with in vitro–in vivo translation. Lab A Chip.

[121] Milani N., Parrott N., Galetin A., Fowler S., Gertz M. (2024). In silico modeling and simulation of organ-on-a-chip systems to support data analysis and a priori experimental design. CPT Pharmacomet. Syst. Pharmacol..

[122] Taylor D.L., Gough A., Schurdak M.E., Vernetti L., Chennubhotla C.S., Lefever D., Pei F., Faeder J.R., Lezon T.R., Stern A.M. (2019). Harnessing human microphysiology systems as key experimental models for quantitative systems pharmacology. Concepts and Principles of Pharmacology: 100 Years of the Handbook of Experimental Pharmacology.

[123] Bowman C.M., Benet L.Z. (2019). In vitro-in vivo extrapolation and hepatic clearance-dependent underprediction. J. Pharm. Sci..

[124] Sager J.E., Yu J., Ragueneau-Majlessi I., Isoherranen N. (2015). Physiologically based pharmacokinetic (PBPK) modeling and simulation approaches: A systematic review of published models, applications, and model verification. Drug Metab. Dispos..

[125] Mehta V., Karnam G., Madgula V. (2024). Liver-on-chips for drug discovery and development. Mater. Today Bio.

[126] Kang S.J., Yoon M.S., Lee J.M., Jo M.J., Jin C.E., Shin D.H. (2026). Recapitulating In Vivo Pharmacokinetics and Size-Dependent Nanomedicine Delivery in a Microfluidic Platform. Adv. Sci..

[127] Rayner C.R., Smith P.F., Andes D., Andrews K., Derendorf H., Friberg L.E., Hanna D., Lepak A., Mills E., Polasek T.M. (2021). Model-informed drug development for anti-infectives: State of the art and future. Clin. Pharmacol. Ther..

[128] Miller P.G., Shuler M.L. (2016). Design and demonstration of a pumpless 14 compartment microphysiological system. Biotechnol. Bioeng..

[129] Wikswo J.P., Curtis E.L., Eagleton Z.E., Evans B.C., Kole A., Hofmeister L.H., Matloff W.J. (2013). Scaling and systems biology for integrating multiple organs-on-a-chip. Lab A Chip.

[130] Hargrove-Grimes P., Low L.A., Tagle D.A. (2021). Microphysiological systems: What it takes for community adoption. Exp. Biol. Med..

[131] Miedel M.T., Varmazyad M., Xia M., Brooks M.M., Gavlock D.C., Reese C., Behari J., Soto-Gutierrez A., Gough A., Taylor D.L. (2025). Validation of microphysiological systems for interpreting patient heterogeneity requires robust reproducibility analytics and experimental metadata. Cell Rep. Methods.

[132] Nahon D.M., Moerkens R., Aydogmus H., Lendemeijer B., Martínez-Silgado A., Stein J.M., Dostanić M., Frimat J.-P., Gontan C., de Graaf M.N. (2024). Standardizing designed and emergent quantitative features in microphysiological systems. Nat. Biomed. Eng..

[133] Pamies D., Ekert J., Zurich M.-G., Frey O., Werner S., Piergiovanni M., Freedman B.S., Teo A.K.K., Erfurth H., Reyes D.R. (2024). Recommendations on fit-for-purpose criteria to establish quality management for microphysiological systems and for monitoring their reproducibility. Stem Cell Rep..

[134] Barr J.T., Lade J.M., Tran T.B., Dahal U.P. (2019). Fraction unbound for liver microsome and hepatocyte incubations for all major species can be approximated using a single-species surrogate. Drug Metab. Dispos..

[135] Francis L.J., Houston J., Hallifax D. (2021). Impact of plasma protein binding in drug clearance prediction: A database analysis of published studies and implications for in vitro-in vivo extrapolation. Drug Metab. Dispos..

[136] Winkler T.E., Herland A. (2021). Sorption of neuropsychopharmaca in microfluidic materials for in vitro studies. ACS Appl. Mater. Interfaces.

[137] Morrison A.I., Sjoerds M.J., Vonk L.A., Gibbs S., Koning J.J. (2024). In vitro immunity: An overview of immunocompetent organ-on-chip models. Front. Immunol..

[138] Stanke-Labesque F., Gautier-Veyret E., Chhun S., Guilhaumou R., French Society of Pharmacology and Therapeutics (2020). Inflammation is a major regulator of drug metabolizing enzymes and transporters: Consequences for the personalization of drug treatment. Pharmacol. Ther..

[139] Miller C.P., Shin W., Ahn E.H., Kim H.J., Kim D.-H. (2020). Engineering microphysiological immune system responses on chips. Trends Biotechnol..

[140] Ekert J., Mohanan S., Deakyne J., Allen P.P., Marshall N., Jeong C., Getsios S. (2020). Integration of the Immune System Into Complex In Vitro Models for Preclinical Drug Development. Biomimetic Microengineering.

[141] Deng S., Li C., Cao J., Cui Z., Du J., Fu Z., Yang H., Chen P. (2023). Organ-on-a-chip meets artificial intelligence in drug evaluation. Theranostics.

[142] Gangwal A., Lavecchia A. (2025). Artificial intelligence in preclinical research: Enhancing digital twins and organ-on-chip to reduce animal testing. Drug Discov. Today.

[143] Wang W., Wang N., Wu Y., Ye Z., Zhao L., Chen X., Ouyang D. (2025). An Integrated AI-PBPK Platform for Predicting Drug In Vivo Fate and Tissue Distribution in Human and Inter-Species Extrapolation. Clin. Pharmacol. Ther..

[144] Casas B., Vilen L., Bauer S., Kanebratt K.P., Wennberg Huldt C., Magnusson L., Marx U., Andersson T.B., Gennemark P., Cedersund G. (2022). Integrated experimental-computational analysis of a HepaRG liver-islet microphysiological system for human-centric diabetes research. PLoS Comput. Biol..

[145] Maass C., Stokes C.L., Griffith L.G., Cirit M. (2017). Multi-functional scaling methodology for translational pharmacokinetic and pharmacodynamic applications using integrated microphysiological systems (MPS). Integr. Biol..

[146] Akbarialiabad H., Seyyedi M.S., Paydar S., Habibzadeh A., Haghighi A., Kvedar J.C. (2024). Bridging silicon and carbon worlds with digital twins and on-chip systems in drug discovery. npj Syst. Biol. Appl..

[147] Aravindakshan M.R., Mandal C., Pothen A., Schaller S., Maass C. (2025). DigiLoCS: A leap forward in predictive organ-on-chip simulations. PLoS ONE.

